# An event-related potentials account of brain predictive coding

**DOI:** 10.1007/s11571-026-10491-7

**Published:** 2026-06-30

**Authors:** Carlos M. Gómez, Antonio Arjona, Francisco J. Ruíz-Martínez, Manuel Muñoz-Caracuel, Vanesa Muñoz, Elena I. Rodriguez-Martinez

**Affiliations:** 1 Human Psychobiology Lab, Experimental Psychology Department, University of Seville, Seville, Spain; 2 Department of Psychiatry, University of Valladolid, Valladolid, Spain; 3 Department of Psychiatry, University Hospital Virgen del Rocio, Andalusian Health Service, Seville, Spain

**Keywords:** Predictive processing, Predictive coding, Brain Bayesian processing, Event-related potentials, Contingent negative variation, P300, Mismatch negativity, Post-imperative negative variation

## Abstract

Predictive coding is a theory that tries to account for how the brain processes in an anticipatory manner the expected stimuli, and reorganizes the underlying neural networks as a consequence of the outcome of predictions: Correct or incorrect. EEG has the advantage of making a continuous and almost instantaneous record of brain activity. The present report summarizes work on Event-Related Potentials (ERPs) and reviews the neural validity of Predictive processing as a mechanism to predict future events, assess the validity of predictions, and then update the probabilities associated with future events. Using two experimental models: predictive tone sequences and central cue Posner paradigms and Bayesian modelling, the report suggests that Contingent Negative Variation (CNV) would be related to prior expectation, Mismatch negativity (MMN) and P300 to Bayesian surprise and/or prediction error, and Post Imperative Negative Variation (PINV) to the assessment of trial outcome in uncertainty situations. The review tends to support predictive coding as a theory consistent with brain operations indexed by ERPs.

## Predictive processing: a unified framework for brain and mind functioning

In recent years, Predictive Processing has emerged as a promising unified approach for studying brain and mind function. It integrates traditionally independent processes such as perception, action, cognition, and emotion into a single theoretical framework (Friston 2005, 2009; Mendonça et al. 2020). The core premise of Predictive Processing is that the brain is fundamentally a prediction machine that continuously generates internal hierarchical models of the world to anticipate sensory inputs. The goal is to minimize prediction errors by either adjusting these models as new information is received or modifying behavior to align with predictions. This optimization enhances the brain’s ability to adapt to environmental demands.

To computationally formalize how the brain engages in predictive behavior, a generative model based on inferential statistics is required. In this context, Bayesian approaches have become one of the most widely used mathematical frameworks for explaining how the brain updates and adjusts its hypotheses based on sensory stimulation (Knill and Pouget 2004; Pouget et al. 2013). Bayes’ Theorem, formulated by English mathematician Thomas Bayes (1702–1761), provides a probabilistic approach for updating the posterior probability of a hypothesis based on prior probabilities and new evidence (empirical data). Mathematically:

1 $$\:P\left(A|B\right)=\frac{P\left(B|A\right)P\left(A\right)}{P\left(B\right)}$$

**Fig. 1:**
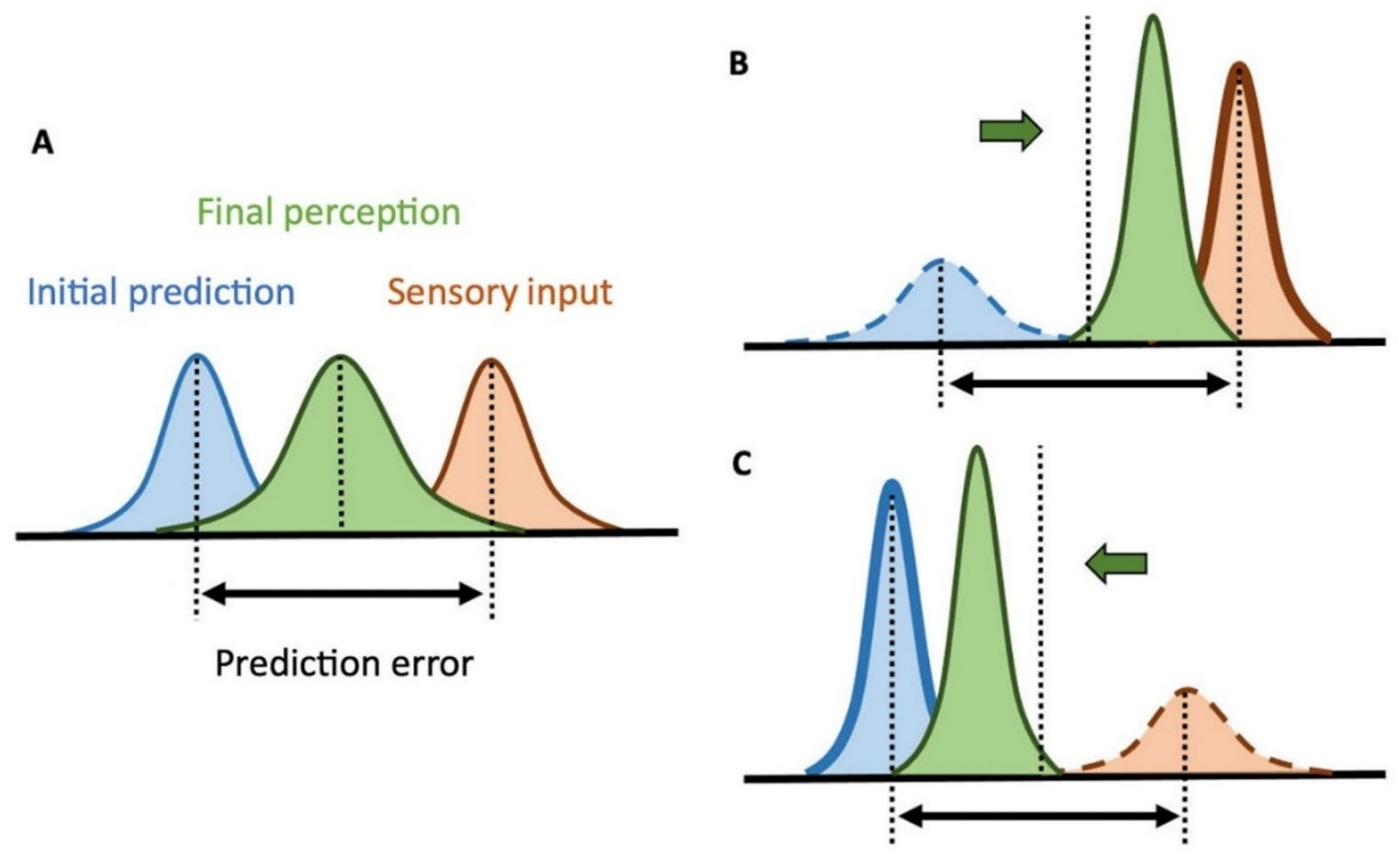
Graphical representation of the Bayesian approach applied to predictive processing. **A** The Gaussian probability distribution that will define the final perception depends on both the distributions of the initial predictions and the sensory inputs. **B** Greater precision assigned to the sensory inputs will cause the final perception to resemble them more closely. **C** Greater precision assigned to the predictions will result in a final perception that is further from the sensory inputs and closer to the initial predictions. Source: adapted from Adams et al. (2013).

In Predictive Processing, the brain’s initial predictions correspond to P(A), while P(B∣A) represents the probability of receiving a sensory input B given that hypothesis A is true. Finally, P(A∣B) reflects the brain’s final perception after updating its initial predictions with new sensory evidence (Fig. 1).

The Bayesian framework not only explains how the brain minimizes prediction errors but also suggests that this process is implemented through a hierarchically organized inferential system. Information flows both upward and downward within the brain’s hierarchy:

- Higher-level brain areas (e.g., prefrontal cortex) generate predictions that travel downward toward lower levels.
- Lower-level areas (e.g., sensory cortices) compare these predictions with actual sensory inputs.
- If a discrepancy (prediction error) occurs, a corrective signal is sent upward to update the predictive model (Rao And Ballard 1999; Friston 2005, 2010; Carbajal And Malmierca 2018).

This hierarchical structure allows the brain to predict both fast, low-level sensory features and slow, high-level abstract regularities (Hohwy 2013).

## The electroencephalogram as a tool for studying predictive coding

Electroencephalography (EEG) and magnetoencephalography (MEG) are two non-invasive techniques that allow the investigation of different aspects of brain activity. Although they have limited spatial resolution, they are particularly well suited for characterizing the amplitude and temporal dynamics of neural responses associated with stimulus processing or task execution. The derived Event Related Potentials (ERPs, recorded with EEG), Event-Related Fields (ERF, recorded with MEG) in the time domain, and the Event-Related Spectral Perturbations (ERSP, recorded from both) are metrics that have been successfully used to analyze the predictive processin hypothesis in the brain. Now we will review some of these EEG-derived results to understand how the brain codes for the different subprocesses related to predictive coding. In this vein, we will attempt to associate certain ERPs with the cognitive operations proposed in predictive processing theory, namely prior probability, prediction error, and Bayesian surprise (Friston et al. 2015; Hohwy 2020; Walsh et al. 2020). The latter two processes contribute to belief updating by revising prior probabilities, which, in an S1–S2 experimental paradigm, correspond to the conditional probability P(S2|S1).

The EEG captures electrical currents generated by the synchronous activity of neuronal populations, primarily within the cerebral cortex. This is due both to their proximity to the scalp and to the open-field spatial configuration of the electrical fields produced by the columnar and parallel organization of the apical dendrites of cortical pyramidal cells. For these signals to be observable at the scalp as ERPs, excitatory and inhibitory postsynaptic potentials must exhibit a high degree of temporal synchronization and parallel spatial directionality. This synchronization and parallel organization are necessary to generate sufficient amplitude to be recorded at a distance and to overcome the high electrical resistance of the skull. However, this technique is inherently limited in its specificity with respect to neural circuitry, as it reflects the algebraic summation of activity from large neuronal populations. Consequently, EEG is not optimal for precise spatial localization. The effects of volume conduction make accurate source localization difficult, and scalp-recorded signals represent the superposition of electrical activity arising from different cortical layers and brain regions. To address these limitations, several methods have been developed to infer the underlying neural sources, including dipole source localization, Low-Resolution Electromagnetic Tomography (LORETA), and minimum-norm estimation, among others. Despite these advances, it remains challenging to directly associate specific ERP components with the activation of precise cortical layers or regions (Luck 2014).

As a result, ERPs are not ideally suited for distinguishing between top-down and bottom-up signals in the brain, except in cases where temporal differences can be exploited (e.g., pre- versus post-target activity). This distinction is particularly relevant in current theories of hierarchically organized predictive coding. In this framework, it has been proposed that superficial layer neurons encode prediction errors, while deep layer neurons encode expectations and generate descending (feedback) predictions (Friston 2005; Friston And Kiebel 2009). Achieving a precise characterization of these hierarchical, bidirectional flows of information—central to Bayesian models of brain function—would require techniques with higher spatial resolution to characterize laminar-specific processing, such as high-field fMRI pushed to its technical limits such as high-field fMRI pushed to its technical limits, for example, using Vascular Space Occupancy (VASO) imaging (Huber et al. 2018), or ultra-high-field 7 Tesla fMRI, which allows the separation of infra-, supra-, and granular cortical layers. Using such approaches, increased activation of infragranular layers has been reported during the perception of illusory Kanizsa figures (Kok et al. 2016). Similarly, Aitken et al. (2020) showed that visually presented stimuli preferentially elicited activity in supragranular layers, whereas responses to stimulus omissions, presumably driven by top-down signals, were stronger in deeper layers. An alternative approach with greater anatomical precision involves animal studies using laminar current source density and local field potential recordings during paradigms designed to probe predictive processing (Pinotsis et al., 2019). Using these techniques, it has been proposed that gamma-band activity (40–150 Hz) reaches peak amplitude in superficial layers II and III, whereas alpha- and beta-band activity (8–30 Hz) peaks in deeper layers V and VI (Bastos et al. 2020). Theta-band activity has also been associated with superficial layers. Although still a matter of debate, some authors have linked theta and gamma oscillations to prediction error signaling in superficial layers, and alpha and beta oscillations to top-down predictions in deeper layers (Bastos et al. 2020). However, other studies have suggested that gamma oscillations may be related to stimulus predictability (Uran et al. 2022). Therefore, the possibility of using ERSPs to infer top-down predictions and prediction errors from electrophysiological signals—as suggested by laminar current source density and local field potential recordings in animals—represents another important advantage of human electrophysiological recordings.

ERPs are characterized by a set of properties that allow them to be distinguished and classified into different components. Each component is defined by a specific combination of features, including latency (measured in milliseconds from stimulus onset to peak amplitude), polarity (positive or negative), experimental sensitivity (the degree to which it is associated with a particular experimental manipulation), and topography (the spatial distribution across the scalp, which provides approximate information about the underlying neural sources) (Donchin et al. 1978a, b). Each of these characteristics corresponds to a distinct role in sensory and/or cognitive processing and allows ERP components to be organized according to shared features. One of the most widely accepted classifications distinguishes between two broad groups of components based on their latency: exogenous (or early) components, occurring within the first 50–150 ms after stimulus onset, and endogenous (or late) components, which occur thereafter. Due to their rapid onset, exogenous components are primarily associated with the early, automatic processing of the physical properties of stimuli, such as sensory modality (e.g., visual or auditory) or intensity. In contrast, endogenous components, with their longer latencies, are linked to higher-order cognitive processing, including the evaluation of stimulus meaning and task-related information processing.

These later components are particularly influenced by psychological factors such as attention, motivation, and arousal. It is important to note that this classification is fundamentally theoretical, as there is no strict latency boundary at which one type of processing ends and another begins. Rather, the influence of stimulus properties and cognitive processes evolves continuously across components, reflecting a dynamic balance between exogenous and endogenous contributions that shifts progressively with increasing latency (Picton 1988). One of the major advantages of ERPs is precisely their ability to provide a temporally resolved analysis of cortical processing. This cannot be achieved with blood-flow-based techniques such as fMRI, which, although potentially capable of differentiating spatially supra- and infragranular layers at the limits of spatial resolution, suffer from poor temporal resolution that may conflate processes occurring at different time points. For instance, ERPs may allow the temporal dissociation of top-down signals occurring prior to target onset from post-target processes such as prediction-error signaling and belief updating—distinctions that are difficult to achieve with slower techniques such as fMRI.

## Contingent negative variation as a proxy for priors

Any successful application of ERPs to predictive coding theory should demonstrate the presence of signals that would detect the presence of a priori processing in the brain. The Contingent Negative Variation (CNV) is one of the most promising signals to capture the a priori signaling of future target stimuli, or in more ecological terms, the most likely next scenario.

The CNV is a slow, late-latency, large-amplitude negative potential associated with expectancy and preparatory processes (Walter et al. 1964; Rockstroh et al. 1982). This component emerges in the interval between the perception of a warning stimulus, which prepares or alerts the individual to the upcoming event, and the appearance of the anticipated stimulus. It has been linked both to sustained attentional engagement during a task and to the preparation of motor responses (Eimer 1993; Gómez et al. 2004). Across multiple studies, this negative shift has been localized in fronto-central and posterior brain regions (Cui et al. 2000; Gómez et al. 2001).

Research employing cue-based paradigms has examined this negative potential in relation to the sensorimotor pre-activation required for successful task performance (Brunia And van Boxtel 2001; Gómez et al. 2001, 2004; Flores et al. 2009; Mento 2013). The classical experimental design for studying the CNV consists of presenting a warning cue (S1) followed, after a relatively short interval, by an imperative or target stimulus (S2). Within this framework, the CNV typically begins to develop approximately 300–500 ms after S1 onset and, depending on the length of the foreperiod, can extend for several seconds. Following S2, some form of response (motor or mental) is usually required to elicit a clear CNV (Tecce 1972). The S1 cue functions as a signal that initiates activation of the neural systems necessary for processing S2 and for preparing the potential motor or cognitive response (Gómez et al. 2003, 2004).

**Fig. 2:**
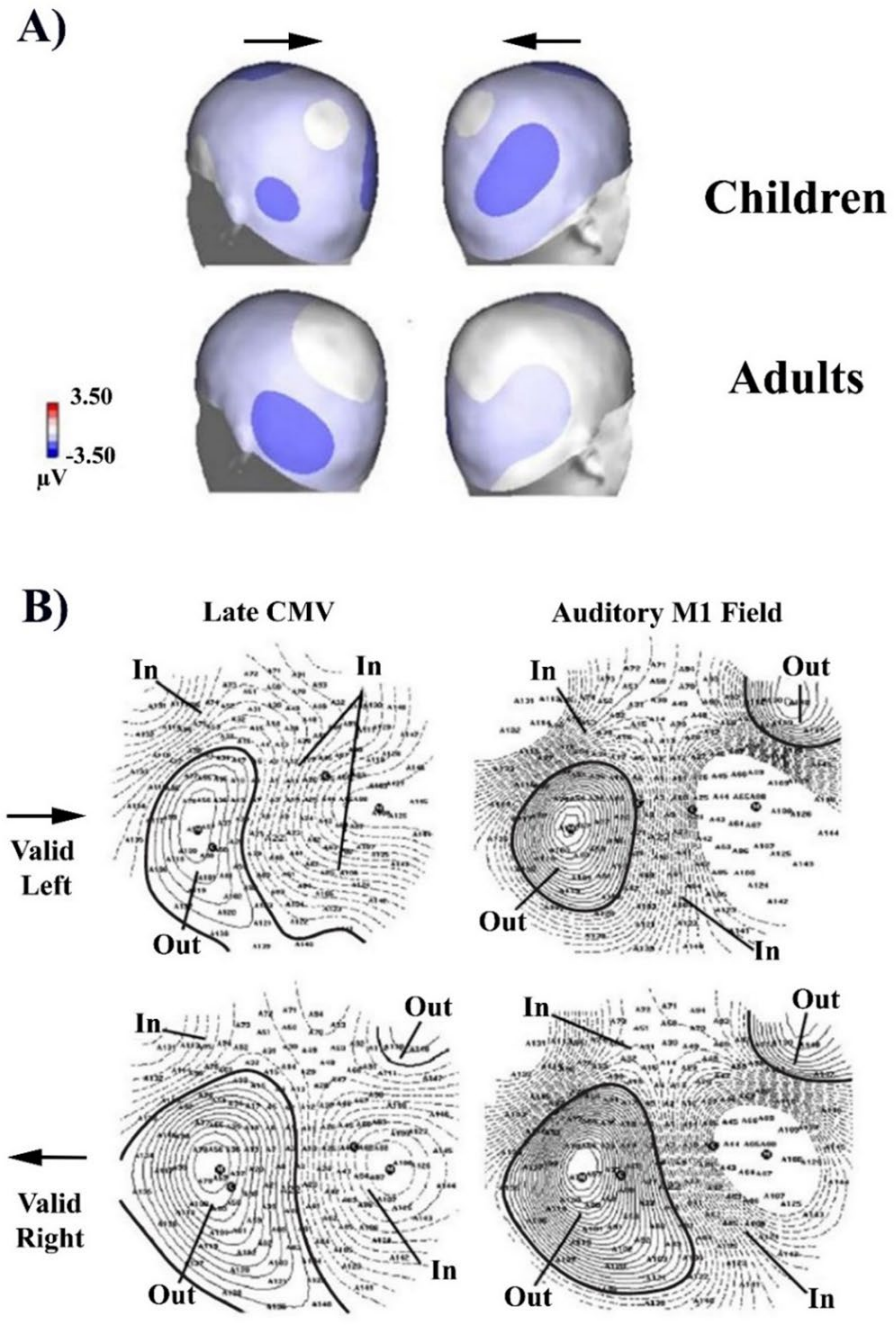
Contralateral cortical activation induced by the central cue. **A** Topographic map of CNV with a maximum in the side contralateral to the cue indicated location. **B** This figure shows the Contingent Magnetic Variation on the left side of the figure, and the auditory M1 field in the right part. In the upper part of the figure are shown the Event-Related Fields when a left cue is presented, and in the lower part when the cue indicates the right side. Notice the similarity between the preparatory and the stimulus processing period for visual (**A**) and auditory (**B**) stimulation (From Flores et al. 2009; Gómez et al. 2004)

From the perspective of the Threshold Regulation Theory, the CNV negativity is interpreted as an increase in neuronal pre-activation mediated by depolarization of the apical dendrites of pyramidal neurons (Rockstroh et al. 1982). Therefore, from the perspective of predictive coding, the cognitive system of the brain should be able to predictively prepare the neural set related to the expected stimulus. In fact, directing the attention by a central cue, in a predictive manner to a spatial location in auditory and visual central cue Posner paradigms, the visual cortex (Fig. 2A; Flores et al. 2009) and the auditory cortex (Fig. 2B; Gómez et al. 2004) showed the activation to the contralateral side to the location indicated by the central spatial cue, suggesting that the cue pre-activated the sensory neural assemblies for accomplishing the task in an anticipatory (predictive of next location) manner.

**Fig. 3:**
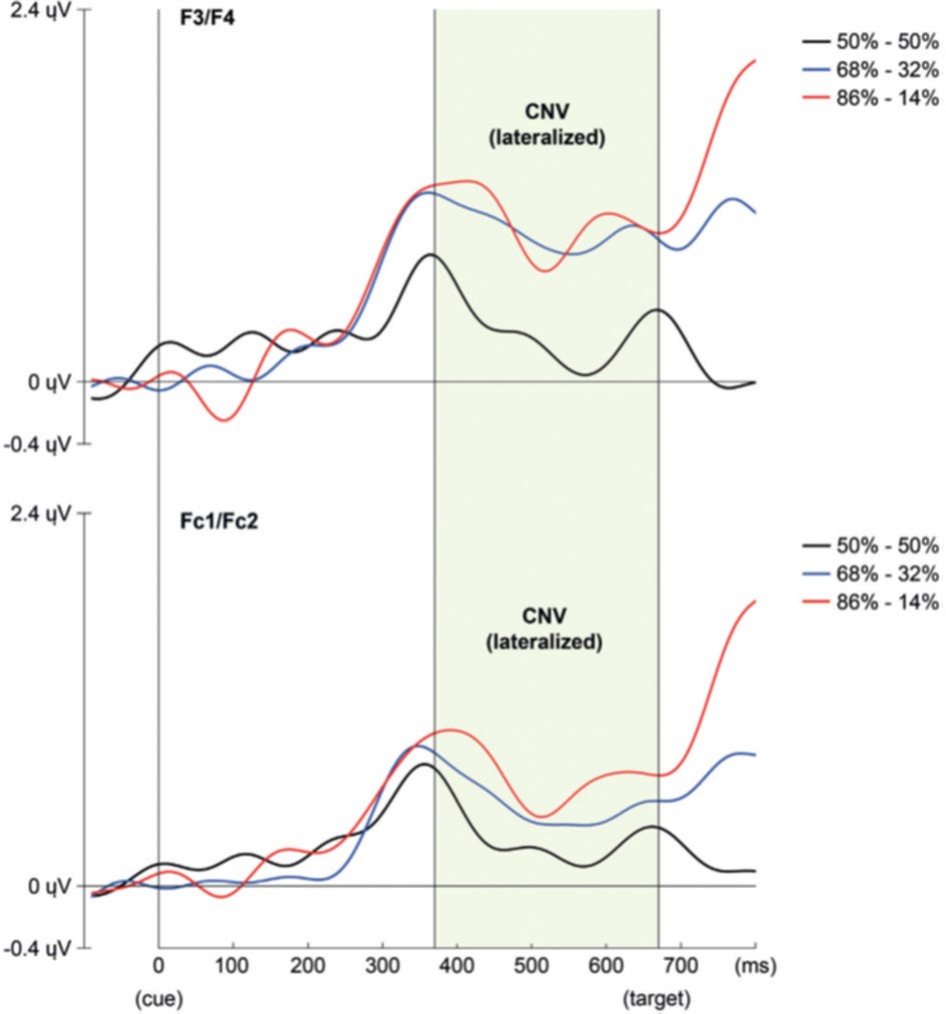
Effects of the type of block on the lateralized component of CNV. The panel shows the amplitude of the lateralized component of CNV induced by the three types of blocks. Note the trend toward higher positivity as the proportion of valid trials per block increases. The lateralized component of CNV was performed with the same method as the so-called Lateralized Readiness Potential (LRP) ((F3-F4) Left arrow − (F3-F4) Right arrow)/2. (From Arjona et al. 2016)

To be consistent with predictive coding, CNV amplitude should depend not only on the sensory modality and the spatially indicated location, but also on the conditional probability that the cue validly predicts the target location, *p*(S2|S1). In line with this view, the lateralized CNV, analyzed using pairs of homologous lateral electrode sites, showed a significant increase in a condition in which cue validity was 86%, compared with a 50% validity block (Arjona et al. 2016). This result suggests enhanced preparatory activity, likely involving not only motor but also sensory processes when the proportion of valid trials is higher. The latter could be interpreted as if contralateral to the cue, the cortex assigns a higher probability to the possible location when the cue has a high validity (Fig. 3). Therefore, two main characteristics of the next stimulus are inferred from CNV, the next target location and the probability that the target stimulus would appear in a given spatial location (prior probability associated to the spatial location). The results shown in Fig. 3 suggest enhanced preparatory activity, likely involving the motor cortex contralateral to the cued side, which is greater when the proportion of valid trials is higher. This finding may be interpreted as indicating that the cortex contralateral to the cue assigns a higher probability to the expected target location when cue validity is high (Fig. 3), thereby leading to increased motor preparation. However, early auditory components (P1/N1) have been shown to be fronto-central and contralateral to the stimulated ear (see Figs. 2 and 3 in Woldorff And Hillyard 1991). Therefore, the lateralized CNV may also include an auditory sensory component, which is difficult to disentangle from the motor contribution. Taken together, the CNV may reflect two key characteristics of the upcoming stimulus: (i) its expected spatial location (with contributions from both motor and auditory cortices), and (ii) the probability that the stimulus will occur at a given location (i.e., prior probability encoded within motor and/or auditory regions). This relationship between lateralized CNV and the spatial and probabilistic allocation of neural activity is consistent with predictive coding theory.

One possible explanation for the increased amplitude of the lateralized CNV in the 86% block is that it reflects an increase in the brain’s baseline activity. In line with this interpretation, Summerfield and de Lange (2014) proposed that changes in baseline activity constitute a mechanism for processing shifts in a priori probabilities. Thus, the lateralized CNV may index such changes, arising from a continuous flow of positive charge into the apical dendrites of pyramidal neurons, leading to modulation of their response threshold, as suggested by the Threshold Regulation Theory (Rockstroh et al. 1982). A more continuous account of CNV as indexing the subjective prior expectation values is presented in the section on sequential analysis. These results of increased CNV in the high validity conditions have been recently replicated by Rabinovich and Telesheva (2025).

However, an alternative interpretation of the CNV, rather than as a reflection of predictability, is the more classical view that CNV indexes the pre-target attentional engagement of a task-related neural set. In fact, the attentional interpretation is supported by the absence of differences between the 86% and 68% cue-validity conditions, suggesting an all-or-none deployment of attention guided by the cue. In the central-cue Posner paradigm, it is difficult to dissociate predictability from attention, and it is possible that these constructs partly reflect different linguistic characterizations of processes that are, if not identical, at least highly interrelated within this paradigm: Prediction and attention. As will be discussed later, CNV amplitude—at least in the context of the central-cue Posner paradigm—appears to align with the Bayesian concept of prior probability, given its relationship with this parameter when applying trial-by-trial Bayesian modeling of prior probability and Bayesian surprise (Gómez et al. 2019; Mathys et al. 2011). Such model takes into account both local (sequential effects) and global (cue–target probabilities within a block of trials) factors. This Bayesian account would imply a continuous evaluation of cue predictability, as indexed by the CNV, rather than a categorical deployment of attention. Further studies are needed to disentangle the respective contributions of attentional and predictive processes—or their interaction—in modulating the CNV amplitude. The possibility of orthogonalizing prediction and attention when investigating the cognitive role of the CNV would be critical for clarifying its functional significance, further than a mere linguistic labelling, as it has been suggested in other contexts (Alink And Blank 2021).

## Mismatch negativity (MMN) in standard-deviant paradigms

The mismatch negativity (MMN) is a pre-attentive response generated between 100-250ms after the presentation of a stimulus, manifesting as a negativity in the fronto-central region of the scalp (Näätänen 1995; Näätänen and Winkler 1999). This component is primarily elicited during the oddball paradigm. The presentation of the deviant stimulus causes a negative N1 response with greater amplitude and a latency similar to the standard, with the difference (obtained by subtracting the voltage from the standard to that of the deviant) resulting in the negative component identified as MMN. It is generated by the unexpected appearance of a deviant auditory stimulus, different from the standard stimulus that the subject expects, which has been previously stored in their echoic memory after being repeatedly presented. However, the standard could be not only a specific sound, but a sequence, or a rule that interrelates them (Lang et al. 1995; Winkler 2007; Escera And Corral 2007; Näätänen et al. 2007; Winkler And Czigler 2012; Paavilainen 2013).

The deviant can occur due to any difference in the physical characteristics of the presented standards (amplitude, frequency, duration, etc.) (Tervaniemi et al. 2000; Ylinen et al. 2006; Novitski et al. 2007), or due to any modification of the pattern that links them, such as the time interval between stimuli, the increase or decrease of the expected frequency or amplitude for a stimulus based on a previously established rule, the absence of an expected stimulus or sound sequence, etc. (Paavilainen et al. 1995, 1998, 1999, 2013, 2018). Furthermore, the deviant can be provoked in passive experimental designs, in which the stimulation does not require attention or any type of response or analysis by the subject (Näätänen et al. 1995, 2007, 2012). Therefore, MMN is considered a pre-attentional component.

This pre-attentive response is considered an indicator aimed at detecting environmental changes in order to manage the number of resources required for information processing, depending on its novelty (Winkler 2007; Winkler And Czigler 2012; Näätänen et al. 2012). To date, the exact role of the brain areas involved in the generation of auditory MMN has not been fully delineated. However, there is a broad consensus on the fundamental role played by the auditory cortex in activating the supratemporal areas as an initial mechanism for detecting and representing pre-attentional patterns in the received stimulation. This region, along with the medial and inferior parts of the frontal cortex, has also been linked to the subsequent formation of the fronto-central negativity that characterizes this component, which is thought to modulate involuntary attention based on the perceived changes in environmental sounds. However, the precise localization of the generators within the frontal cortex remains to be specified, as well as the supposed contribution of the hippocampus and thalamus (Näätänen et al. 2007; Deouell 2007; Sussman 2007; Paavilainen 2013).

There are fundamentally two hypotheses to explain the psychophysiological mechanism that generates the MMN. One of them proposes that the greater amplitude of the deviant stimulus, compared to the standard, would simply be caused by a basic process of neural habituation, so that the continuous repetition of the standard would result in a reduction of the response generated by the areas involved in its processing. In this case, the appearance of the deviant would generate a greater amplitude compared to the standard or, rather, an unattenuated amplitude, since this stimulus would not be habituated due to the differences it presents compared to the standard (May et al. 1999; May And Tiitinen 2004, 2010).

The other theory attempting to explain the origin of this component is called the Hypothesis of Regularity Violation and is framed within predictive coding (Winkler 2007). According to this theory, MMN would be the product of the prediction error generated when the internal representation or expectation of the next stimulus is contradicted. The MMN relationship with predictive coding arises from the fact that MMN not only appears when the physical characteristics of the expected stimuli are violated, but also when the abstract relationships established between them are contradicted, which cannot be explained solely by a process of habituation (Paavilainen et al. 1999, 2018; Korzyukov et al. 2003; Winkler 2007; Winkler And Czigler 2012; Ruiz-Martínez et al. 2021, 2022). The Näätänen et al. proposal (2007) is more related to the formation of a model in sensory memory that is being compared with new stimuli, i.e., a process of deviant detection rather than an active prediction as in the Hypothesis of Regularity Violation (Winkler 2007).

Also referring to the predictive coding theory, Wacongne et al. in 2012, proposed a neurophysiological model for the generation of MMN, based on the interrelationship of four neural layers, each with a distinct role. According to this model, there would be a thalamic layer where the characteristics of both the standard and the deviant are represented, along with three additional cortical layers responsible for processing the prediction error, the generative internal model, and the stored sensory information in memory, respectively. Thus, the prediction error would be generated in a specific layer by integrating the activity produced by both the thalamic layer and the layer responsible for generating predictions, which in turn would receive inputs from the layer responsible for storing the recently perceived stimuli in the memory trace. In this model, the thalamic layer would send excitatory signals to the layer responsible for prediction error, whereas the predictive layer would send inhibitory signals. Thus, the prediction error generating the MMN would be the result of situations where the tonic inhibition of the predictive layer cannot cancel the phasic excitatory input from the thalamic layer. The authors also propose that if the prediction is based on more abstract rules, the model would require the participation of a new, hierarchically superior layer of neurons sensitive to the complex features of the stimulation pattern, which would provide feedback to the prediction error layer.

**Fig. 4:**
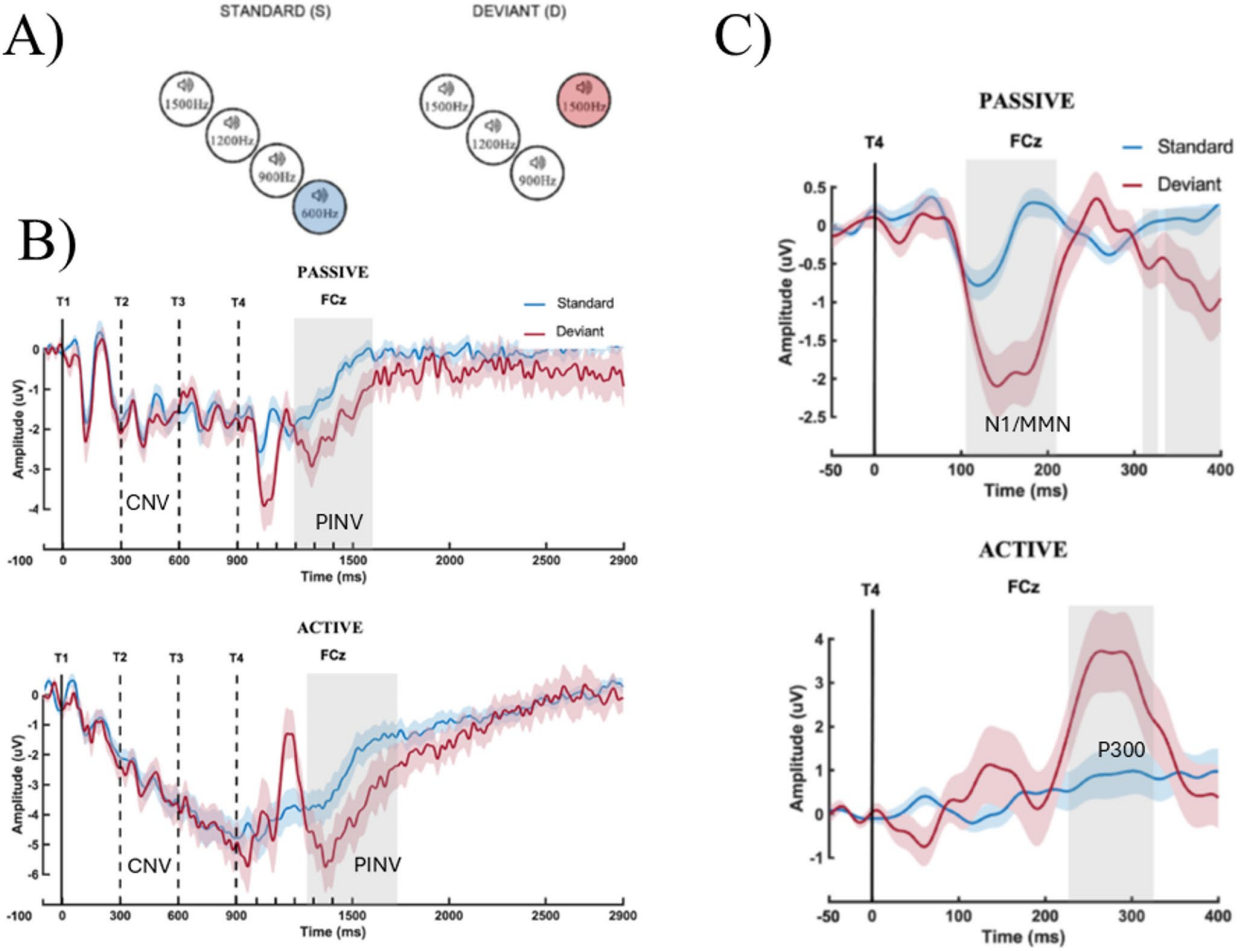
Tones sequences paradigm and corresponding elicited ERSPs. **A** Sequence of frequency tones in the first order condition (low complexity) for Standard trials (S), and deviant trials (D). **B** Statistical comparison (gray area) computed for the long-time window (post-T1) between the standard and deviant trials in the FCz electrode for the passive and active conditions. Note the similar dynamics of the PINV in the passive and active conditions, and the higher amplitude of CNV in the active condition. **C** Statistical comparison (gray area) computed for the short-time window (post-T4) between the standard and deviant trials in the FCz electrode for the passive and active conditions. Note the N1/MMN increase in the passive deviant and the increase of P300 in the active deviant conditions. Please notice that the MMN and P300 were preceded by a CNV, with a higher amplitude in the active condition. Event-Related Potentials are indicated. CNV: Contingent Negative Variation. N1/MMN; Mismatch negativity overimposed on the N1; P300; PINV: Post Imperative Negative Variation. T1 to T4 indicate the time of presentation of the four auditory tones. (From Muñoz-Caracuel et al. 2024)

In recent studies, a passive sequential experimental paradigm was particularly designed for understanding generative prediction and prediction errors by means of tone sequences (Ruiz Martínez et al. 2021, 2022; Muñoz-Caracuel et al. 2024). The MMN protocol consisted of sequences of four tones with either increasing or decreasing frequencies (counterbalanced across subjects), all exhibiting the same frequency progression except for deviant stimuli, which occurred randomly in the final position (Fig. 4A). The simplest, first-order paradigm was composed of a single sequence of sounds, whereas the more complex, second-order paradigm comprised five different sequences, with different frequencies in each sequence. From a Bayesian-processing perspective, extracting patterns in these two experimental protocols would require different levels of abstraction. Using this sort of paradigms (Fig. 4B and C), it was possible to record an MMN to deviant trials in the passive condition (no required responses), which, given the low predictability of the deviant stimulus, could be considered as a sign of prediction error (Fig. 4C). For the active condition (different responses were required to deviants and standards), the tone frequency unpredictability of the last tone was indexed by a P300 component, rather than by MMN. The latter result suggested that attentional and pre-attentional prediction errors were indexed by P300 and MMN, respectively, although possibly the MMN in the active condition was obscured by the high amplitude P300 in the active condition (Muñoz-Caracuel et al. 2024).

The MMN elicited by violations of the standard trials in the second-order paradigm, in which presented tone frequencies were constantly changing across the experiment, would support the view that this component plays a role in prediction error, rather than being merely the result of a psychophysiological process associated with the adaptation theory (Garrido et al. 2009). According to the adaptation account, the reduced response to the standard stimulus is attributed to neural habituation caused by its repeated presentation, whereas the deviant stimulus, being less frequently encountered, would elicit a larger amplitude due to the absence of habituation (May et al. 1999; Jääskeläinen et al. 2004; May And Tiitinen 2004, 2010).

The reason why auditory MMN generation in the complex paradigm cannot be satisfactorily explained by an adaptation process lies in the microcolumnar organization of the primary auditory cortex, arranged according to the frequencies it processes, which gives rise to a tonotopic map. This organization has been verified using EEG (Bertrand et al. 1988), intracranial electrodes (Howard et al. 1996), MEG (Romani et al. 1982; Pantev et al. 1988), and blood-flow imaging (Lauter et al. 1985). Such tonotopic organization would prevent habituation to the sounds forming the standard trials of the second-order complex paradigm (frequencies of tones changing constantly), because the wide range of frequencies and the limited number of repetitions would make habituation difficult: the same microcolumns are stimulated only a few times, and these occurrences are spaced apart in time.

The auditory MMN obtained in the second-order paradigm of this experiment therefore supports previous studies proposing that this component is sensitive not only to differences in the physical characteristics of the presented sounds, but also to violations of abstract rules extracted from complex tone sequences (Wacongne et al. 2012; Tervaniemi et al. 1994; Ono et al. 2017; Winkler 2007). In the present study, the pattern underlying the experimental paradigm was based on the variant frequency progression (either ascending or descending) present in the tone sequences; thus, the deviant stimulus constituted a violation of the directional rule and consequently elicited an MMN.

The proposal that the MMN observed in the four sequential tones (S1–S4) is not primarily driven by habituation, but rather by a violation of the predicted frequency of the final tone, is further supported by the finding that the N1 elicited by S1 and S4 shows higher amplitude than the N1 evoked by S2 and S3, in both standard and deviant conditions. The reduced amplitude observed for S2 and S3 can be attributed to habituation effects. In contrast, the relatively higher N1 amplitude for S4 in the standard condition, compared to S2 and S3, is more plausibly explained by the allocation of increased neural resources to process a stimulus at a position where a rule violation is expected. This interpretation is further supported by the presence of a developing CNV during the S1–S4 interval, consistent with its proposed role in task-related sensory anticipation (Gómez et al. 2004). Disentangling the violation of a predicted signal from surprise as a computational role for MMN, however, would require more sophisticated experimental paradigms, such as that employed by Feuerriegel et al. (2021), in which oddball faces with varying probabilities are embedded within sequences of frequently presented base stimuli. While these findings appear robust in the visual modality, replication of similar paradigms would be necessary before extending this interpretation to the auditory domain. Finally, in the four-tone paradigm, the difference between standard and deviant responses computed for change trials (SD and DS) is greater than for no-change trials (SS and DD), suggesting a continuous updating of expectancy probabilities assigned to tones within the sequence (Ruiz-Martínez et al. 2022). This result, further developed in the section on the sequential N1 component, supports a probabilistic interpretation of the MMN in the auditory modality, rather than an all-or-none effect.

However, the adaptation hypothesis can still be supported (Jääskeläinen et al. 2004), not only because of the evidence in its favor but also due to the difficulty of refuting it. However, the four-tone protocol inherently emphasizes the predictive component by design. Moreover, the increased amplitude of the fourth tone, regardless of whether it is standard or deviant, compared to the second and third tones argues against a simple adaptation account. Nevertheless, if both prediction violation and adaptation hypotheses remain plausible, particularly in the context of abstract rule processing, the MMN sources identified in frontal cortical regions may represent the most likely neural substrates for either the detection of abstract rules or adaptation to standard regularities. This interpretation is consistent with previous findings that have localized MMN-related activity to frontal areas (Garrido et al. 2009; Muñoz-Caracuel et al. 2024).”

In summary, the MMN results suggest an involvement of the primary auditory cortex, and possibly the frontal cortex, in the generation of predictive error (Friston, 2009), indicating that it is an index of the comparison between the predicted features related to physical features or abstract rules and the features of the current stimulus, in accordance with the regularity-violation theory associated with this component, still in a passive condition in which attention was diverted from the auditory stimulation (Winkler 2007). The involvement of the primary auditory cortex as a source for MMN in passive conditions was supported by an experiment using functional Near Infrared Spectroscopy (fNIRS) during a predictive MMN paradigm (Muñoz-Caracuel et al. 2024). When some active responses were required, the MMN can be obliterated by the high P300 amplitude, which in this case would be the clearer ERP for indexing regularity violation detection, while fNIRS suggested a frontal involvement (Muñoz-Caracuel et al. 2024).

The study of the Event-Related Spectral Power (ERSP) during passive paradigm described above showed that in addition to the MMN (Ruíz-Martínez et al. 2025), a series of modulation of oscillatory components were ascribed to prediction error (early frontocentral theta), and the increase in late beta and gamma would be related to the integration of updated probabilities for standard and deviants to next trials. The latter argument was supported by previous studies using reward paradigms, which have proposed that these brain rhythms index the relationship between attention and reward, with higher activity observed for unexpected events (Marco-Pallarés et al. 2015).

## P300 component: a possible index of prediction error and/or beliefs updating

The P3 or P300 component is a positive polarity potential, with a latency range typically between 250 and 500 milliseconds after the presentation of a stimulus to which the subject must assess and/or respond. The first report of the P300 potential dates back approximately 50 years (Sutton et al. 1965). The so-called Oddball paradigm was crucial in relating the amplitude of this component to the probability of occurrence of the target stimulus as well as the relevance of the task (Donchin et al. 1978a, b; Pritchard 1981). In this paradigm, the amplitude of the P300 component would indicate the neural activation related to the updating of the mental representation of the target stimulus. Thus, when a novel or unexpected stimulus appears, the attentional mechanism would be activated to initiate an evaluation process that occurs in working memory to update the information about the target stimulus’s representation (Donchin 1981; Heslenfeld 2003).

Although the P300 is easily replicable through paradigms such as the Oddball or Posner’s Central Cue Paradigm, there is still no clear understanding of how and why this potential occurs. On the other hand, it has also been observed that the variation in the interval between stimuli affects the amplitude of the P300 (Polich et al. 1994, 2007), as well as the involvement of memory in the task (Polich 2007; Azizian et al. 2007). In this sense, theories linking the P300 with processes of working memory updating and attentional processing are the most widely accepted due to their versatility in adapting to various results (Donchin et al. 1986, 1988).

The P300, since its inception, has been divided into two subcomponents: P3a and P3b. The subcomponent known as P3a would present a more fronto-central topography, with a peak latency in the range between 250 and 280 milliseconds after the appearance of the target stimulus. This would be a potential related to the attentional processing of the stimulus (especially orientation) and the possible novelty associated with its arrival (Friedman et al. 2001). Meanwhile, P3b would refer to a more posterior and late positivity, appearing approximately 300 milliseconds after the target stimulus (ranging from 250 to 500 milliseconds). This subcomponent has been very useful for studying the processing of information in different cognitive processes. Regarding localization, various studies of patients with brain lesions have shed light on the possible neural sources of the P300. It has been reported that lesions in the frontal lobe cause a decrease in the amplitude of P3a, but not P3b, indicating that the latter has a more posterior origin. Furthermore, the reduction of P3a has been observed after lesions in both the lateral prefrontal cortex and the orbitofrontal cortex (Løvstad et al. 2012). On the other hand, lesions in the temporo-parietal junction have been linked to decreased amplitude in both components. In sum, P3a seems to have a greater dependence on frontal areas, while P3b seems to have a more posterior origin (Bledowsky et al. 2004).

The relationship between the P300 and the probability of the received stimuli has been observed since the earliest theoretical proposals. According to Duncan-Johnson and Donchin (1977, 1982), and Squires et al. (1976), the P300 amplitude increases depending on how unexpected the stimulus generating it is. In fact, it has been related to the amount of information conveyed by the stimulus, as a function of the a priori expectancy. Squires et al. (1976), proposed an equation in which the amplitude of P300 was a function of three terms: (i) a decaying memory (M) component which provides that recent presentations of a given stimulus increases the expectancy that it will recur, but the influence of each presentation decays over trials; (ii) a term (Alt) which accounts for the expectancy generated by sequences of alternating stimuli (e.g., AaAa), and (iii) the most directly related to the Bayesian predictive coding (the P term), which accounts for a priori probability of the stimulus.

2 $$\:ER{P}_{amplitude}=b0+b1*M+b2*Alt+b3*P$$

However, a limitation of classical Oddball paradigms is that the a priori probabilities of standards and deviants must be implicitly estimated by the participant through statistical learning of their relative frequencies. An alternative approach has been implemented by directing attention by a cue that has a certain probability to indicate the target location, in both auditory (Gómez et al. 2004) and visual locations (Mangun And Hillyard 1991; Flores et al. 2009). These kinds of experiments use a central cue, following a Posner-type protocol. As shown in Fig. 2, the cue elicits preparatory activation in the contralateral visual cortex (Fig. 2A) and auditory cortex (Fig. 2B), indicating that attentional orienting can bias the participant’s expectancy in a top-down manner.

This methodology allows examination of how the P300 responds to the *validity* (confirmation of the predicted target) or *invalidity* (violation of the prediction) of the central cue. Increased P300 amplitudes were observed following invalid cues (Mangun And Hillyard 1991; Flores et al. 2009). These results provide further evidence that these potentials are associated with prediction-error processing, that is, the discrepancy between expected and actual events (Kolossa et al. 2015; Seer et al. 2016), suggesting that the P300 operates as a prediction-error signal when the cue-induced expectation is violated (Friston 2005; Gómez et al. 2019). This working memory updating process may originate from the incongruence between the information provided by the cue and the actual stimulus, allowing for a reevaluation of the cue’s reliability. In summary, changes in cue validity (from valid to invalid) render invalid outcomes more unexpected, increasing the need for prediction-error analysis and updating of the inferred cue–target contingency. These approaches support early proposals that P300 amplitude is inversely related to target expectancy (Squires et al. 1976) and further indicate that it may constitute one of the neural signals indexing prediction error (Friston 2005).

A deeper analysis of the role of P300 as an index of prediction error would be obtained by varying cue validity. This was accomplished by Arjona et al. (2016) in an experiment in which the cue validity probability was set to 50%, 68%, and 86%. The effect of the cue on reaction times (shorter RTs in valid trials compared with invalid trials) increased as the proportion of valid trials per block rose (50% < 68% < 86%), indicating that higher proportions of valid trials enhanced the sensory/motor preparation induced by the cue, and vice versa. The percentage of incorrect responses in invalid trials, relative to valid trials, also increased as the proportion of valid trials per block became larger. This pattern indicates a greater tendency to produce impulsive, incorrect responses when the number of valid trials was higher. This strong behavioral effect was reflected in the ERPs.

**Fig. 5:**
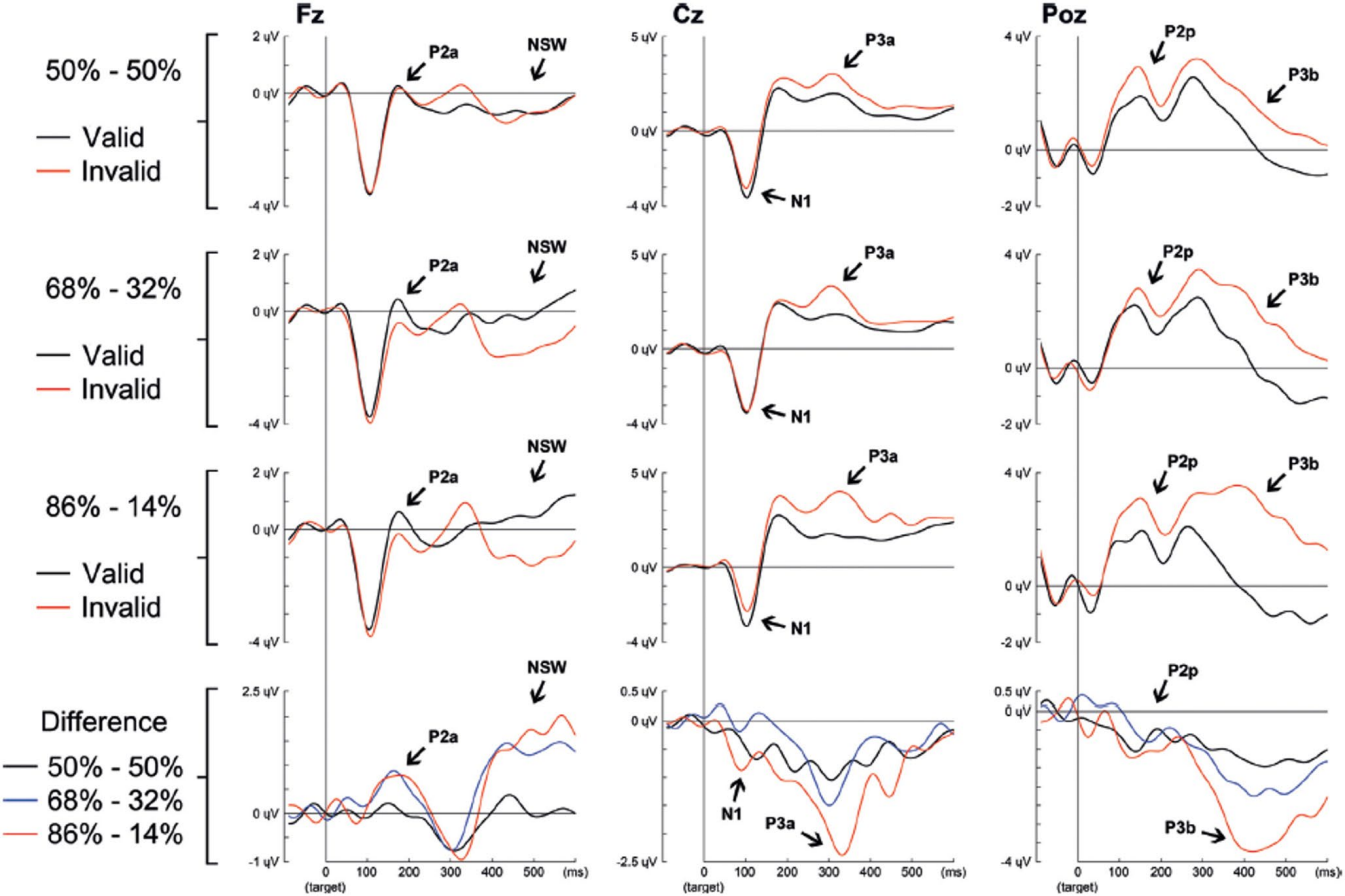
Post-target ERPs potentials in a central posner paradigm, with different probability validity. The figure shows the post-target ERPs (N1, P2a, P2p, P3a, P3b, and NSW) for valid and invalid trials, as well as the corresponding difference waves (valid minus invalid) across the three block types. The block types (50%, 68%, 86%, and the block difference) are displayed from top to bottom. From left to right, the figure presents data from three midline electrodes: Fz, Cz, and POz. The ERP components are labeled within each panel: P2a and NSW at Fz; N1 and P3a at Cz; and P2p and P3b at POz. Notably, (i) N1 and P2a amplitudes are larger in valid than in invalid trials, whereas (ii) P2p, P3a, P3b, and NSW amplitudes are larger in invalid than in valid trials (From Arjona et al. 2016)

In the above described paradigm, a series of ERPs changed amplitude as a function of validity. The P2a component showed greater amplitude in valid than in invalid trials, with this difference being larger in the blocks containing 86% and 68% valid trials than in the 50% block (Fig. 5). This effect likely reflects enhanced post-target attentional processing in valid trials as their proportion increases, given that the P2a component is associated with frontal mechanisms regulating selective attention (Potts et al. 1998). Previous studies have reported that P2a amplitude does not vary as a function of the sensory modality of the target stimulus or the specific response required in the task, suggesting that this component is more closely related to the relevance of the stimulus or the attention directed toward it (Potts et al. 1998, 2004; Potts And Tucker 2001). In addition, this frontal component appears to be closely related to the so-called Frontal Selection Positivity (FSP) (Kenemans et al. 1993; Makeig et al. 1999), which has been linked to the selection of relevant stimulus features and the preparation of the appropriate response. All these previous results suggest that the cue probability modulated the intensity of this process as a function of cue probability.

The P3a and P3b (Fig. 5) components showed larger amplitudes in invalid trials relative to valid trials; however, only the P3b exhibited an increasing difference between conditions as the proportion of valid trials per block increased (Arjona et al. 2016). The latter study showed greater P3b amplitude in invalid trials compared with valid trials, and this difference increased as the proportion of valid trials per block became larger. This effect suggests that invalid trials trigger an updating of the cue’s value as a predictor of the target, and that the magnitude of this updating is larger when invalid trials are less frequent (because the target becomes more unexpected). In other words, the enhancement of the P3b in invalid trials likely reflects the trial-by-trial working-memory updating process, or beliefs updating, required to incorporate new information about what has occurred (Donchin And Coles 1988; Sommer et al. 1998; Arjona et al. 2014). This effect would then be a consequence of the a priori probabilities as suggested by Squires et al. (1976), and reflected in Eq. 2 by the term a priori probability (P), but making the term P explicit in the experiment by including a cue with changing probability. The increase of P300 amplitude invalidity effect in the high validity condition has been recently replicated (Rabinovich And Telesheva 2025). In fact, there was a linear relationship between the amplitude of the CNV induced by the cue and the amplitude of the invalidity effect when the target is presented (Arjona et al. 2016). Suggesting that the P300 invalidity effect is a function of the a priori probability assigned to the cue for predicting the target.

Within the framework of the Bayesian brain hypothesis (Friston, 2009), this increase in P3b amplitude for invalid trials would be associated with the processing of the so-called *prediction error*, as well as with the updating of the conditional probability p(S2|S1) linking the cue (S1) to the target stimulus (S2) (Gómez and Flores 2011). In line with this interpretation, the positive correlation observed between the modulation of lateralized CNV on a given trial and the P3b supports the Bayesian view of the P3b as a component associated with evaluating the match between the prediction (indexed by the CNV) and the actual location of the target stimulus (Arjona et al. 2016). However, as indicated several times across the manuscript, a possible relationship of P300 modulation with attention is difficult to discard in the type of experiments here described.

The Negative Slow Wave (NSW) (Fig. 5) component also showed a higher amplitude in invalid trials with respect to valid trials, with higher invalid effects in the high validity blocks (Arjona et al. 2016). It can be suggested that this negativity may be similar and possibly functionally homologous to the so-called PINV in sequences of tones (Ruíz-Martínez et al. 2022; Muñoz-Caracuel et al. 2024). This interpretation is supported by similar latency, topography, and reactivity to unexpected events. Furthermore, both are preceded by CNV developing before the unexpected event. In this case, the NSW would have, like the PINV (see below), the same processing role proposed by Elbert et al. (1970), which suggested that the PINV (and NSW) would be induced when expectations are contradicted from previous experiences, and would participate in the assessment of presented stimuli contingencies.

### Post imperative negative variation

Interestingly, the MMN obtained in the passive paradigm, and the P300 obtained in the active paradigm, and presented in the previous sections, were followed by a long-lasting negativity (Fig. 4B) (Ruíz-Martínez et al. 2022; Muñoz-Caracuel et al. 2024), which was identified as a Post Imperative Negative Variation (PINV).

In an S1-S2 paradigm, the CNV generated by the S1 typically returns to baseline immediately following the onset of S2. Under certain conditions, however, this recovery is prolonged, giving rise to a PINV, which generally exhibits a prefrontal maximum (Diener et al. 2009). In the experiment described above, a late and slow frontally distributed negative was recorded and categorized as a PINV (Ruíz-Martínez et al. 2021, 2022; Muñoz-Caracuel et al. 2024). Factors known to elicit this potential include uncertainty regarding the correctness of a response, difficulty integrating a response into a symbolic framework, no control over an aversive stimulus, and the need to re-evaluate ambiguous contingencies (Dongier 1969; Kathman et al. 1990; Klein et al. 1996; Bender et al. 2006). The PINV then seems to play a role in predictive coding, which, to our knowledge, has not previously been described in the literature. This finding would support the proposal by Elbert et al. (1970), who suggested that the PINV is generated whenever the outcome of a subject’s response contradicts expectations formed from prior experience, although this argument can also be extended to experimental conditions in which no overt response is required (Ruíz-Martínez et al. 2022).

### Sequential effects

In the present review on the central cue Posner paradigm, we are dealing with the concept that the different phases of predictive coding in a trial can be defined by means of ERPs and ERSPs. The a priori probability would be indexed by the CNV, the assessment and updating of the a priori values to become the *a posteriori* probability would be related to the P3b amplitude (and theta), but also to the PINV, while the transmission of information to the next trial would be possibly related to the presence of long-range beta and gamma activities. The four tones paradigm would add MMN as an index of prediction error. But a crucial consequence of predictive coding would be the possibility of detecting the effect of a trial in priors and prediction errors of the next trial. Therefore, behavioral sequences and ERPs analyses would permit to recreate the consequences of the updating probabilities in a given trial.

### Sequential behavioral results

In the central cue Posner paradigm, it would be possible to observe the updating of the probabilities associated with the outcomes of the sequences of previous trials. Accordingly, two-trial sequences ending with a valid trial (V) — valid–valid (VV) and invalid–valid (IV) — showed reduced reaction times compared with sequences ending with an invalid trial (I), namely invalid–invalid (II) and valid–invalid (VI) (Arjona and Gómez 2011). The two-trial sequences exhibited a clear pattern of increasing reaction times (VV < IV < II < VI). Of particular interest is the case II < VI RTs, which suggests that reducing the predictive power of the cue improves the RTs in invalid trials. Moreover, the sequences of three trials also showed this dependency of the trial outcome on previous history, and RTs of sequences followed the pattern VVV < IVV< IIV.

The analysis of errors also showed the presence of sequential effects. Regarding errors, the most notable finding was the inverse pattern between incorrect responses (high percentages in VI sequences and low percentages in VV sequences) and anticipatory responses (predominantly in VV sequences and very few in VI sequences). Incorrect responses showed relatively short reaction times, indicating that most errors resulted from premature responding. The previous results on sequential dependency have been termed as the inter-trial validity/invalidity sequential effects (Arjona et al. 2014), and it has been recently replicated (Parisi et al. 2026).

This anticipatory tendency, responsible for both anticipations and incorrect responses, can be interpreted as arising from an interaction between endogenous information (which biases the response toward the cued side) and exogenous information (derived from the target stimulus). In the case of anticipations, these two sources of information are congruent. In contrast, for incorrect responses, the endogenous information triggers an erroneous response before the target stimulus is fully processed. A similar interpretation has been given to the express saccades, which landed in an intermediate position between the expected and the presented target position (Delinte et al. 2002).

The Bayesian inference framework proposed by Friston (2009) is consistent with the results previously described. The invalidity of the prediction made in each trial would reduce the credibility of the cue as a predictor of the target, and the p(target|cue) would be calculated and reevaluated trial-by-trial. These processes would modify the RTs and accuracy as described above. But then, some neurophysiological signals should index the presence of these behavioral sequential effects, embedded in the logic of changing the a priori values of the predicted next stimuli. Two different approaches have confirmed the presence of neurophysiological signatures of the sequential effects: N1 and PINV, on the one hand; and CNV and P3 components, on the other hand.

### The N1 sequential effects

**Fig. 6:**
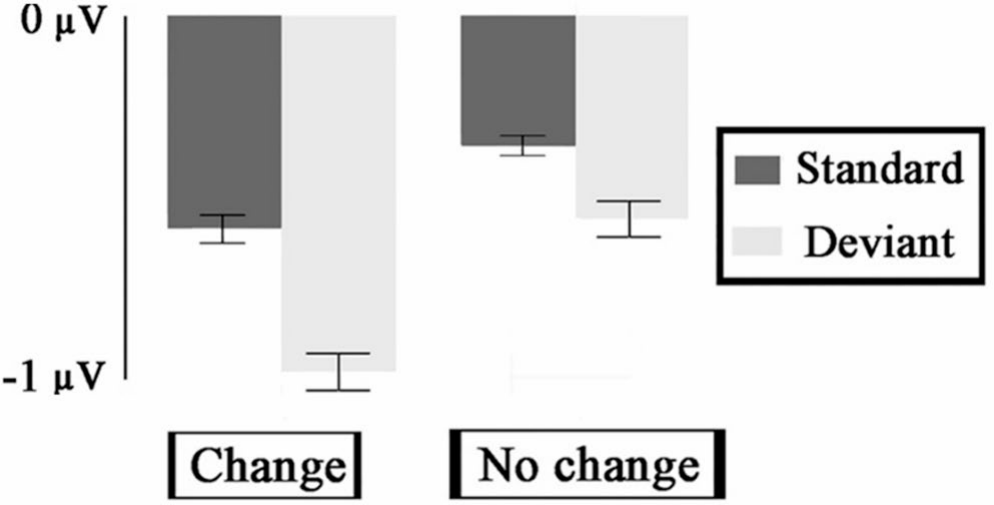
Sequential N1 effects. Amplitude of the N1 to change (different outcome with respect to the previous trial) and (no change, same outcome with respect to the previous trial). Notice that the amplitude of N1 is higher for both standard and deviant trials in the condition of change (From Ruíz-Martínez, 2022)

The comparison of the N1 obtained in a sequence in which two consecutive contiguous sequences were terminated by the same type of outcome (No change), vs. the case in which a trial was followed by a different type of trial (change), induced an N1 higher in the change with respect to the no change condition (Fig. 6). In the four-tone paradigm, the difference between standard and deviant responses computed for change trials (SD and DS, computing S and D in the second element of the dyad) is greater than for no-change trials (SS and DD), suggesting a continuous updating of expectancy probabilities assigned to tones within the sequence (Ruiz-Martínez et al. 2022). This result, supports a probabilistic interpretation of the MMN in the auditory modality, rather than an all-or-none effect, given the continuous updating. This pattern of N1 modulation suggests that its amplitude depends on the outcome of the preceding trial (standard or deviant) and may therefore index trial-by-trial predictability (Ruíz-Martínez et al. 2021, 2022).

The increased negativity for trials in which a different outcome than the previous trial occurs also increases the amplitude of the PINV. These findings suggest that both N1 and PINV are sensitive to the recent history of stimulus sequences, allowing the nervous system to internally categorize each trial as a “standard” or a “deviant” based on whether it repeats or differs from the preceding trial. This pattern indicates that the predicted termination of a stimulus sequence is continuously updated as a function of the previous trial’s outcome. The idea that only two stimuli are sufficient to establish a new abstract rule has also been proposed in the context of MMN responses (Haenschel et al. 2005; Bendixen et al. 2007). Additionally, N1 amplitude is known to be modulated when participants determine the timing of auditory stimulus presentation, showing reduced amplitude under predictable conditions (Martikainen et al. 2005). Taken together, the modulation of N1 by prior stimulus sequences situates auditory processing within a predictive coding framework, in which the brain continually updates its generated predictions as a function of trial outcomes (Bendixen et al. 2007; Winkler and Czigler 2012; Wacogne et al. 2012; Lieder et al. 2013).

### Sequential ERPs effects in the central cue posner paradigm

The CNV would correspond to the neural activation of the cortical areas supposedly required for processing the target stimulus, and this activation would be based on the conditional probability assigned to the cue—p(target|cue)—as updated by the P3b response elicited on the previous trial (Gómez et al. 2009; Chennu et al. 2013; Arjona et al. 2014).

**Fig. 7:**
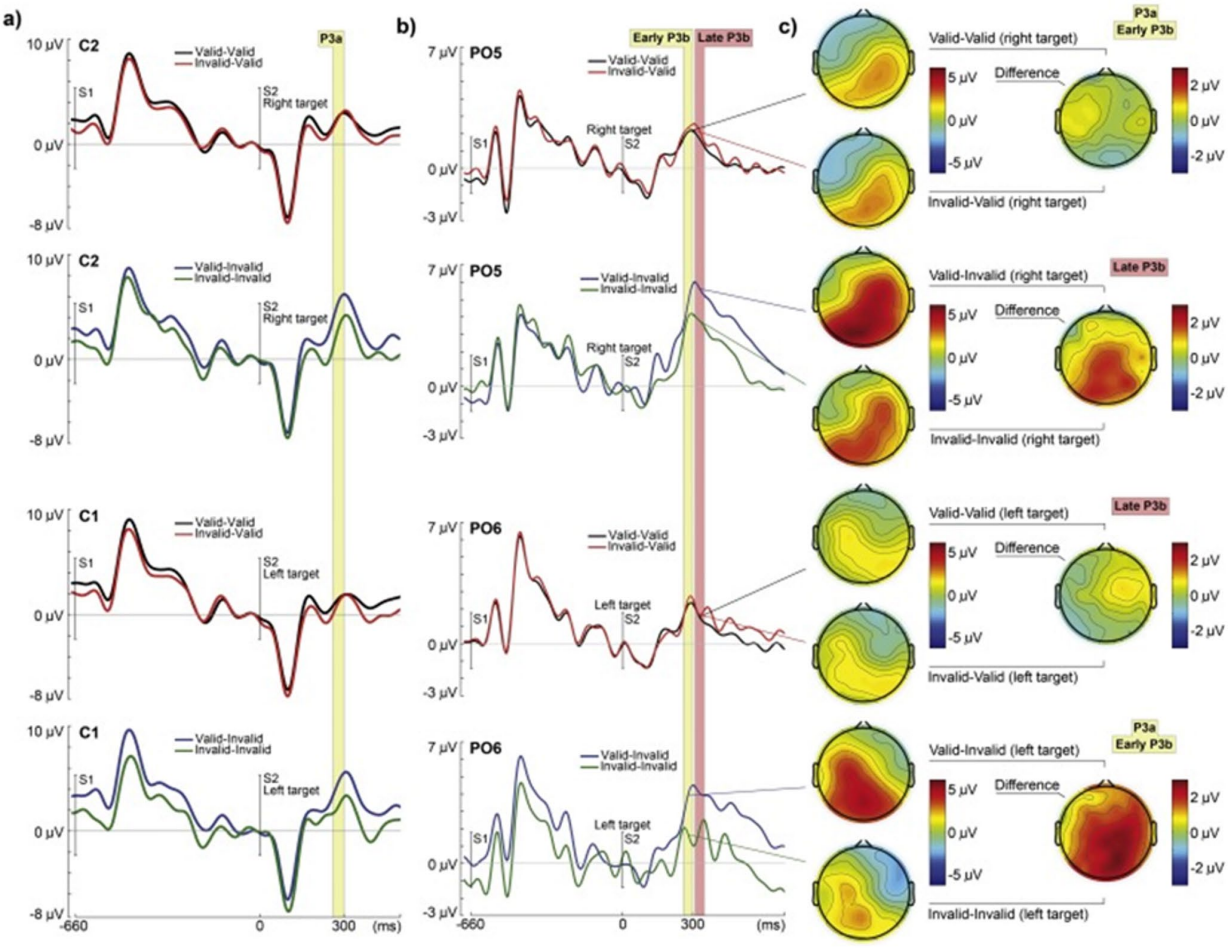
Sequential effects in the CNV P3a and P3b components. Waveforms and topographies indicate that P3a and P3b (early and late) components exhibit greater positive amplitudes in invalid trials preceded by valid trials (VI) compared with invalid trials preceded by invalid trials (II). **a** Show the P3a waveforms. Also, the CNV when the previous trial was invalid presented a lower amplitude than the CNV preceded by a valid trial, once corrected for the baseline (not corrected in the figure for better appreciation of P3b effects). **b** Shows the early and late P3b wave forms, and panel **c** presents the corresponding topographical maps for both components (From Arjona et al. 2017)

This idea of preparatory evaluation has led to the proposal that we are immersed in a cognitive cycle (Gómez and Flores 2011), in which a priori probabilities would be established, indexed by the CNV, and an evaluation of the error made in the prediction would be indexed by the P300 component. This evaluation would imply a change in the a priori expectancy induced by the cue–target association. In fact, the amplitude of the CNV has been shown to be dependent on the outcome of the previous trial (Arjona 2014; Arjona et al. 2014, 2017). As more valid trials precede the current trial, the amplitude of the CNV increases. At the same time, as the number of valid trials increases, a higher P300 amplitude is observed when an invalid trial occurs (Fig. 7; Gómez et al. 2009; Arjona et al. 2017).

### Continuous sequential change of a priori and surprise parameters through Bayesian modelization

All the previous sections have tried to evaluate the relationship of different ERPs to different processes related to predictive coding. But this process should occur in a non-stop, continuous manner. A growing consensus suggests that the human brain functions as a prediction machine. It learns statistical patterns and forms internal generative models, and it constantly uses these models to produce predictions that shape both perception and action (Friston 2010; Friston et al. 2015).

An effective way to understand how the brain extracts and encodes statistical regularities during cognitive tasks is to conduct a model-based computational analysis of participants’ behavioral and neural data (Daw 2011). Studies using this approach have identified several Bayesian-derived parameters that can serve as regressors for fMRI or EEG analyses. These include:

Predictive surprise, which captures the subjective information content, or surprise, associated with an observed event (Shannon 1948).

Bayesian surprise, which reflects the degree of belief updating triggered by a new observation (Baldi And Itti 2010).

Prior expectation, defined as the expected probability of a particular event before it is observed. In trial-by-trial analyses, note that the posterior expectation at trial *T* naturally becomes the prior expectation at trial *T + 1*.

This approach was followed using a model that extracted the prior expectation and Bayesian surprise, and tested the quantitative relationships between ERPs and the Bayesian estimations of prior expectation and Bayesian surprise, on a trial-by-trial basis. This approach was followed by data from a central cue Posner paradigm (50%, 68%, and 86% of validity in different blocks) (Arjona et al. 2016). Single-trial prior expectation and surprise parameters were inferred from participants’ behavior using a Bayesian learning model (HGF, Mathys et al. 2011) that was previously validated in a Posner task (Vossel et al. 2014). The *prior expectation* and *Bayesian surprise* parameters inferred by the model were then correlated with ERP signals recorded from the same participants and trials.

**Fig. 8:**
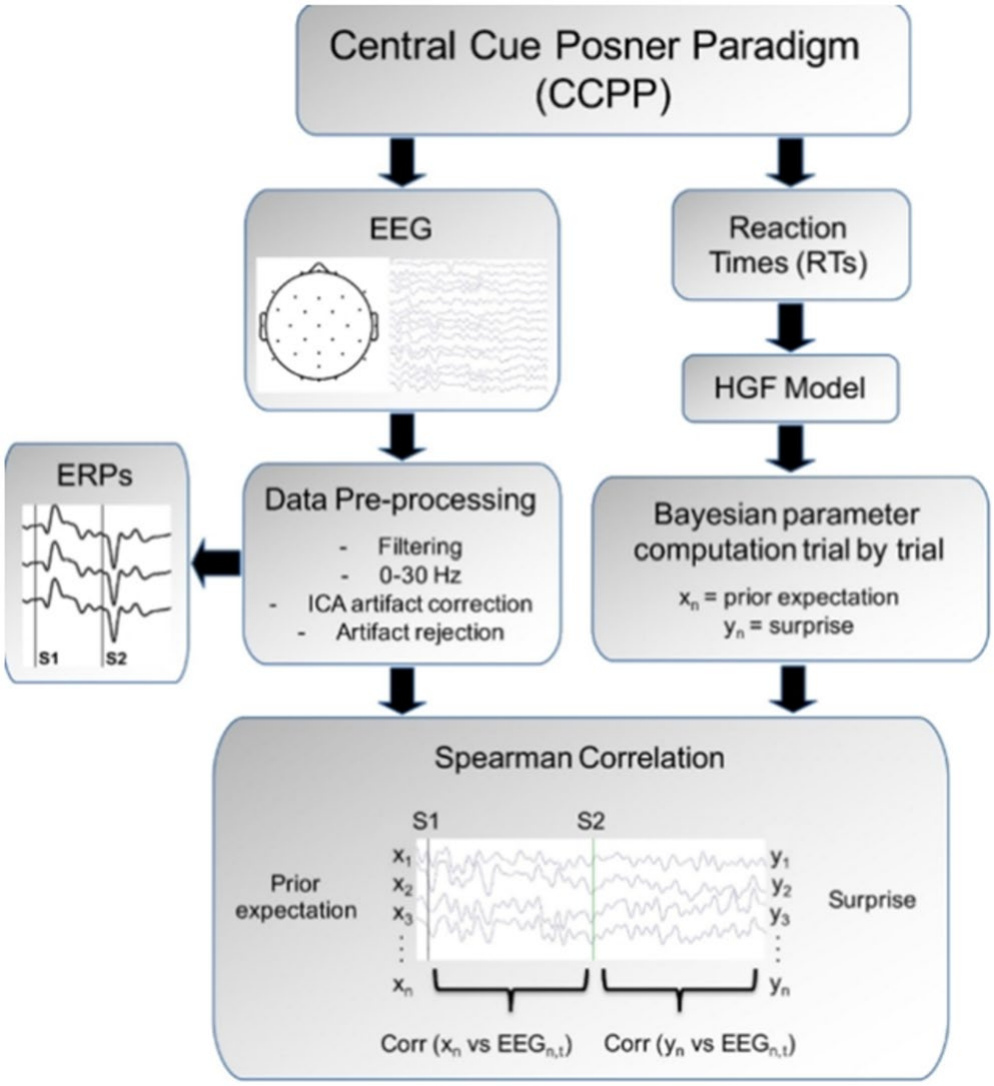
Bayesian modelling and analysis pipeline. Participants performed a Central Cue Posner Paradigm while reaction times and EEG data were recorded. The Hierarchical Gaussian Filter (HGF) model was used to derive two trial-wise parameters: prior expectation (**Xₙ**) and Bayesian surprise (**Yₙ**). After standard EEG pre-processing, event-related potentials (ERPs) elicited by the arrow cue (S1) and the auditory target were extracted (ERP statistical procedures described in Arjona et al. 2016). Finally, trial-by-trial correlations between the HGF parameters (Xₙ and Yₙ) and the EEG signals were computed. (From Gómez et al. 2019)

**Fig. 9:**
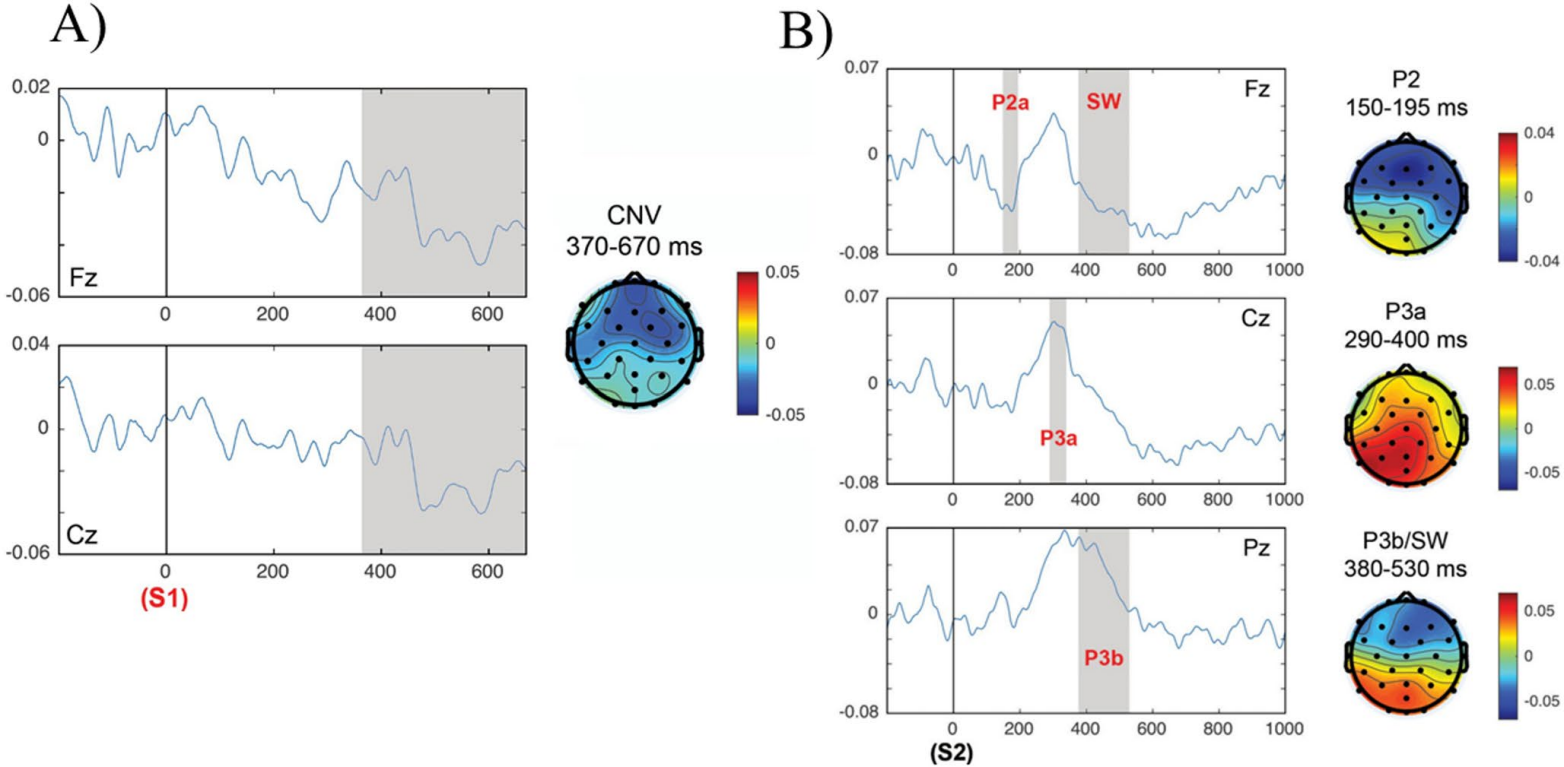
Correlations of Bayesian parameters with single-trial EEG in a central cue posner paradigm. **A** Significant (gray area) Spearman correlation between CNV and the prior expectation parameter, and **B** of post-target ERPs with the Bayesian surprise parameter. (From Gómez et al. 2019)

The data were analyzed following the pipeline shown in Fig. 8, and permitted establishing the windows of significant correlations between the parameters extracted from the model and the ERPs (Fig. 9).

The results indicated that, when examined on a trial-by-trial basis, the EEG signal recorded during a Central Cue Posner task reflects key parameters of Bayesian inference, prior expectation and Bayesian surprise, as estimated by the Hierarchical Gaussian Filter (HGF) model. Prior expectation signals were decoded shortly after the cue, during the CNV period (Fig. 9), whereas the surprise parameter emerged following the target onset at the characteristic latency of the P3a and P3b (Fig. 9), a component traditionally linked to surprise and information gain (Kolossa et al. 2013, 2015; Kopp et al. 2016; Seer et al. 2016).

P2a, considered analogous to the visual FSP, a component associated with the processing of task-relevant stimuli during the transition from selecting relevant features to selecting appropriate responses (Kenemans et al. 1993; Makeig et al. 1999; Potts et al. 2004), was also related to the surprise parameter. Given its negative correlation may reflect that the P2a process confirms the outcomes, situations in which the incoming evidence aligns with prior expectations, thereby facilitating efficient response selection.

Previous work has demonstrated that components of the late positive complex encode Bayesian parameters, with Bayesian surprise reflected in the P3a and predictive (Shannon) surprise reflected in the P3b (Kolossa et al. 2015; Kopp et al. 2016; Seer et al. 2016). As discussed above, the surprise derived from the HGF model constitutes a subjective Bayesian probability: it is generated from a participant-specific generative model whose parameters are estimated from each individual’s responses, rather than from an objective representation of task contingencies. We found this surprise parameter to correlate with voltage latencies in both the P3a and P3b components. During the P3a latency window, the correlation maps revealed a central–posterior distribution, indicating a mixed contribution of P3a and P3b. Importantly, electrodes typically associated with P3a showed significant positive correlations, suggesting that P3a activity is linked to the cognitive processing of Bayesian surprise (Kolossa et al. 2015; Kopp et al. 2016; Seer et al. 2016; Higashi et al. 2017). This correlation extended into the posterior P3b latency window, implying that neural processes underlying P3b generation are also involved in the computation of surprise. Together, these findings suggest that surprise may not be computed in a single step; rather, its neural signature may be distributed across multiple regions, supporting diverse cognitive consequences.

The negative frontal slow wave showed a significant correlation with surprise. Although this component appears within the same time window as the P3b and the positive slow wave , it has a distinct neural origin (Løvstad et al. 2012). Psychophysiologically, this negative slow wave has been proposed to reflect the effort required to reorient attention following distractors, similar to the reorientation negativity (Wetzel and Schröger 2014). Its negative correlation with the surprise parameter suggests that unpredictable stimuli engage frontal networks responsible for redirecting attention toward the next most probable event. However, given the similar latency and topography of this negative component with respect to the PINV, it could also be possible that NSW indexes the assessment of the cue invalidity to facilitate the priors updating in a situation of uncertainty (Elbert, 1970; Ruiz-Martinez et al. 2022; Muñoz-Caracuel et al. 2024).

In sum, trial-by-trial correlations between the prior expectation and surprise parameters derived from the HGF model, based on behavioral data from a Central Cue Posner task, and the EEG signals recorded from the same participants on the same trials were obtained. These findings add to a growing body of evidence indicating that the human brain continuously updates the key variables of Bayesian inference during task performance. Although it is certainly possible to account for these effects in terms of attentional mechanisms (Alink And Blank 2021), this perspective may also point to a strong commonality between attention and predictive coding in brain function. In this sense, their relationship may be analogous to that proposed for attention and conscious experience: while they can be dissociated under certain conditions, they most often co-occur in natural settings.

### Some remarks about predictive processing in language

Although not in the scope of the present review, which is devoted to more basic processes, language seems to be one of the cognitive domains in which predictive coding would be more relevant, given the high conditional probabilistic dependence of the different elements on the language structure, from the succession of phonemes or words, to semantic predictions. Individual differences in statistical learning have been linked to abilities such as sentence comprehension, processing of complex syntactic structures, and both lexical and oral language skills (Evans et al. 2009; Misyak And Christiansen 2012; Mainela-Arnold and Evans 2014). N400 and P600 components have been widely studied within the framework of Bayesian processing to evaluate the statistical learning model of language development, often using sentences with unexpected or incongruent elements (e.g., the cool water burned him) (Kutas And Hillyard 1984; van Herten et al. 2005; León-Cabrera et al. 2017; Wang et al. 2023; Michaelov et al. 2024). Of particular interest is the N400 component, whose amplitude is related to the cloze probability of the sentence.

The components described in the present report correspond to general ERP components such as CNV, N1/MMN, P2, P3a, P3b, and PINV. In contrast, language-related potentials exhibit a higher degree of specificity, as they are associated with distinct aspects of language processing: the N400 reflects the activation of lexical–semantic representations and the retrieval of semantic features not already pre-activated by context; the ELAN is linked to prediction errors in syntactic structure; the LAN is associated with responses to unexpected morphological structure; and the P600 is typically related to violations of structural expectations. In the case of the P600, parallels with the P3b have been proposed, particularly in relation to the detection of incongruities and memory updating processes. The linguistic and predictive processing meaning of these components has been extensively reviewed by Leon-Cabrera et al. (2024). However, the relationship between language-related ERP components and predictive coding is not limited to post-stimulus processing. It likely begins prior to the presentation of the linguistic target. In this context, there is growing interest in anticipatory negativities, such as pre-target prediction negativity in sentence processing and pre-activation negativity (PrAN). These components may correspond to a more fundamental role of the CNV, instantiated within language-processing networks, in generating linguistic quantitative cue–target predictions.

One particular case in which the deficits in statistical learning as primary generators of the priors is the case of Developmental Language Disorder (DLD). Since tracking statistical patterns is essential for language development, difficulties in this ability have been proposed as a key factor underlying language impairments, especially in children diagnosed with DLD (Evans et al. 2009; Hsu And Bishop 2011; Ullman And Pierpont 2005). Experimental research on statistical learning often examines how individuals detect dependencies in language. These dependencies can occur at different levels, such as word segmentation or grammatical structure. In word segmentation tasks, participants hear continuous streams of syllables in which some syllable pairs occur more frequently than others, helping them infer likely word boundaries (Saffran et al. 1997). In contrast, artificial grammar learning studies expose participants to sequences governed by hidden rules or patterns, including nonadjacent dependencies or rule-based structures, without explicitly informing them of these underlying structures (Gómez 2002; Gómez & Gerken, 1999).

On average, individuals diagnosed with DLD show a general cognitive deficit in the implicit detection of statistical regularities in language across different levels (Ullman And Pierpont 2005; Evans et al. 2009; Hsu And Bishop 2011; Hsu et al. 2014), similar to findings observed in the visual domain (Lum et al. 2012, 2014; Obeid et al. 2016). Also, there is growing evidence that interventions grounded in statistical learning principles can support language development in DLD children. These approaches focus on helping children extract patterns from the language they receive, rather than relying on explicit instruction or repetition alone. Plante et al. (2014) found that children made greater progress in using grammatical morphemes when these were presented across a wide range of examples, allowing them to detect underlying regularities.

DLD subjects have shown linguistic ERPs impairment, although these effects were not always explicitly interpreted in terms of statistical learning (Bishop And McArthur 2005; Shafer et al. 2005, 2007; Datta et al. 2010; Kujala And Leminen 2017; An et al. 2022). For some ERP components, the link to statistical learning is more direct. This is the case for the N400 (Kutas And Hillyard 1984). Accordingly, delayed or absent N400 responses in individuals with DLD have been interpreted as reflecting a failure to develop semantically driven prediction error signals (Shafer et al. 2005; Sabisch et al. 2006; Haebig et al. 2018). In addition, a reduction in the Late Positive Component (LPC), which is associated with post-lexical reanalysis and semantic integration, has also been reported in DLD (Haebig et al. 2018). Language processing also involves predictive syntactic information, violations of which elicit ERP components such as the Early Left Anterior Negativity (ELAN) and the P600. Impairments in the development of these components in response to unexpected syntactic and/or semantic violations have therefore been interpreted as evidence for deficits in predictive coding mechanisms in DLD (Fonteneau And van der Lely 2008; Royle And Courteau 2014; Purdy et al. 2014; Haebig et al. 2017). All these results described suggest that language seems to follow predictive mechanisms and assessment of the inferred predictions.

## Conclusions

**Table 1:** Auditory event-related potentials and their possible role in predictive coding

Component	Latency, & topography	Psychological function	Typical paradigm	Possible predictive coding role
CNV	Pre-target; Slow negative; fronto-central	Expectancy, preparation	S1–S2 warning-imperative	Predictions; top-down anticipatory signal (P(S2|S1)
P1	~ 50 ms; +; fronto-central	Early sensory processing	Passive/Active tones	Initial feedforward sensory input
N1	~ 100 ms; −; fronto-central	Stimulus detection	Passive/Active tones	Early prediction error (mismatch with expected sensory input)
MMN	~ 150–250 ms; −; fronto-central	Automatic deviance detection	Auditory oddball	Canonical prediction error signal (violation of sensory regularities)
N2	~ 200–350 ms; −; fronto-central	Conflict/novelty	Go/No-Go	Higher-level prediction error; conflict between competing models
P2	~ 150–250 ms; +; central	Selection of relevant stimulus features	Discrimination	Confirmation of current model
P3a	~ 250–300 ms; +; fronto-central	Attention orienting	Novelty oddball	Bayesian surprise
P3b	~ 300–600 ms; +; parietal	Context updating	Auditory oddball	Model updating; belief revision in response to prediction error
PINV	Slow negative; fronto-central	Post-response evaluation	S1–S2 tasks	Evaluation of prediction outcomes in ambiguous situations; updating action-related expectations

When considered collectively, the brain responses reviewed for the Central Cue Posner, and Sequential Tone paradigms suggest that participants continuously generate predictions about target features and their associated probabilities, with the CNV serving as a key neural marker of anticipatory processing. Prediction error computations, reflected in components such as MMN, PINV, and P300, occur on a trial-by-trial basis and support the updating of prior expectations, thereby maintaining accurate estimates of the conditional probabilities of upcoming events given the current context. Table 1 summarizes schematically the ERPs components here reviewed and its possible role in predictive processing. The attribution of the ERP components discussed here, based on our results as well as those reported by other groups (Carbajal et al. 2018; Chennu et al. 2013; Elbert et al. 1970; Garrido et al. 2009; Gómez et al. 2019; Hohwy 2013; Kolossa et al. 2013 And 2015; Kopp et al. 2016; Winkler, 2007), suggests a close relationship between the MMN/P300 as correlates of prediction error and beliefs updating, the CNV as an index of the conditional prior probability p(S2|S1), and the PINV as reflecting a final reassessment of the relationship between S1 and S2 under conditions of ambiguity. However, these interpretations should be regarded as tentative and not as implying a strict one-to-one correspondence between specific predictive coding operations and individual ERP components.

## Data Availability

No datasets were generated or analysed during the current study.

## References

[CR1] Adams RA, Stephan KE, Brown HR, Frith CD, Friston KJ (2013) The computational anatomy of psychosis. Front Psychiatry 4:47. 10.3389/fpsyt.2013.00047 23750138 10.3389/fpsyt.2013.00047 PMC3667557

[CR2] Aitken F, Menelaou G, Warrington O, Koolschijn RS, Corbin N, Callaghan MF, Kok P (2020) Prior expectations evoke stimulus-specific activity in the deep layers of the primary visual cortex. PLoS Biol 18(12):e3001023. 10.1371/journal.pbio.3001023 33284791 10.1371/journal.pbio.3001023 PMC7746273

[CR3] Alink A, Blank H (2021) Can expectation suppression be explained by reduced attention to predictable stimuli? NeuroImage 231:117824. 10.1016/j.neuroimage.2021.117824 33549756 10.1016/j.neuroimage.2021.117824

[CR4] An WW, Nelson CA, Wilkinson CL (2022) Neural response to repeated auditory stimuli and its association with early language ability in male children with Fragile X syndrome. Front Integr Nuerosci 16:987184. 10.3389/fnint.2022.987184 10.3389/fnint.2022.987184 PMC9702328 36452884

[CR5] Arjona A, Gómez CM (2011) Trial-by-trial changes in a priori informational value of external cues and subjective expectancies in human auditory attention. PLoS ONE 6(6):e21033. 10.1371/journal.pone.0021033 21698164 10.1371/journal.pone.0021033 PMC3116879

[CR6] Arjona A, Gómez CM (2014) Sequential effects in the central cue posner paradigm-on-line bayesian learning. In: Mangun GR (ed) Electrophysiology of attention and cognition. Elsevier, Amsterdam, pp 45–57

[CR10] Azizian A, Polich J (2007) Evidence for attentional gradient in the serial position memory curve from event-related potentials. J Cogn Neurosci 19(12):2071–2081. 10.1162/jocn.2007.19.12.2071 17892393 10.1162/jocn.2007.19.12.2071 PMC2748728

[CR7] Arjona A, Escudero M, Gómez CM (2014) Updating of attentional and premotor allocation resources as function of previous trial outcome. Sci Rep 4:4526. 10.1038/srep04526 24681570 10.1038/srep04526 PMC3970123

[CR8] Arjona A, Escudero M, Gómez CM (2016) Cue validity probability influences neural processing of targets. Biol Psychol 119:171–183. 10.1016/j.biopsycho.2016.07.001 27430935 10.1016/j.biopsycho.2016.07.001

[CR9] Arjona Valladares A, Gómez González J, Gómez CM (2017) Event related potentials changes associated with the processing of auditory valid and invalid targets as a function of previous trial validity in a Posner’s paradigm. Neurosci Res 115:37–43. 10.1016/j.neures.2016.09.006 10.1016/j.neures.2016.09.006 27713025

[CR11] Baldi P, Itti L (2010) Of bits and wows: A Bayesian theory of surprise with applications to attention. Neural Netw 23(5):649–666. 10.1016/j.neunet.2009.12.007 20080025 10.1016/j.neunet.2009.12.007 PMC2860069

[CR12] Bastos AM, Lundqvist M, Waite AS, Kopell N, Miller EK (2020) Layer and rhythm specificity for predictive routing. Proc Natl Acad Sci USA 117(49):31459–31469. 10.1073/pnas.2014868117 33229572 10.1073/pnas.2014868117 PMC7733827

[CR13] Bender S, Becker D, Oelkers-Ax R, Weisbrod M (2006) Cortical motor areas are activated early in a characteristic sequence during post-movement processing. NeuroImage 32(1):333–351. 10.1016/j.neuroimage.2006.03.009 16698286 10.1016/j.neuroimage.2006.03.009

[CR14] Bendixen A, Roeber U, Schröger E (2007) Regularity extraction and application in dynamic auditory stimulus sequences. J Cogn Neurosci 19(10):1664–1677. 10.1162/jocn.2007.19.10.1664 18271740 10.1162/jocn.2007.19.10.1664

[CR15] Bertrand O, Perrin F, Echallier J, Pernier J (1988) Topography and model analysis of auditory evoked potentials: Tonotopic aspects. En Pfurtscheller, G., & Lopes da Silva, F.H. (Eds.) *Functional Brain Imaging*, 75–82. Hans Huber: Toronto, ON, Canada

[CR16] Bishop DV, McArthur GM (2005) Individual differences in auditory processing in specific language impairment: a follow-up study using event-related potentials and behavioural thresholds. Cortex 41(3):327–341. 10.1016/s0010-9452(08)70270-3 15871598 10.1016/s0010-9452(08)70270-3 PMC1266051

[CR17] Bledowski C, Prvulovic D, Hoechstetter K, Scherg M, Wibral M, Goebel R, Linden DE (2004) Localizing P300 generators in visual target and distractor processing: a combined event-related potential and functional magnetic resonance imaging study. J Neurosci 24(42):9353–9360. 10.1523/JNEUROSCI.1897-04.2004 15496671 10.1523/JNEUROSCI.1897-04.2004 PMC6730097

[CR18] Brunia CH, van Boxtel GJ (2001) Wait and see. Int J Psychophysiol 43(1):59–75. 10.1016/s0167-8760(01)00179-9 11742685 10.1016/s0167-8760(01)00179-9

[CR19] Carbajal GV, Malmierca MS (2018) The Neuronal Basis of Predictive Coding Along the Auditory Pathway: From the Subcortical Roots to Cortical Deviance Detection. Trends Hear 22:2331216518784822. 10.1177/2331216518784822 30022729 10.1177/2331216518784822 PMC6053868

[CR20] Chennu S, Noreika V, Gueorguiev D, Blenkmann A, Kochen S, Ibáñez A, Owen AM, Bekinschtein TA (2013) Expectation and attention in hierarchical auditory prediction. J Neurosci 33(27):11194–11205. 10.1523/JNEUROSCI.0114-13.2013 23825422 10.1523/JNEUROSCI.0114-13.2013 PMC3718380

[CR21] Cui RQ, Egkher A, Huter D, Lang W, Lindinger G, Deecke L (2000) High resolution spatiotemporal analysis of the contingent negative variation in simple or complex motor tasks and a non-motor task. Clin Neurophysiol 111(10):1847–1859. 10.1016/s1388-2457(00)00388-6 11018502 10.1016/s1388-2457(00)00388-6

[CR22] Datta H, Shafer VL, Morr ML, Kurtzberg D, Schwartz RG (2010) Electrophysiological indices of discrimination of long-duration, phonetically similar vowels in children with typical and atypical language development. Journal of Speech, Language, and Hearing Research 53(3):757–777. 10.1044/1092-4388(2009/08-0123) 20530387 10.1044/1092-4388(2009/08-0123)

[CR23] Daw ND (2011) Trial-by-trial data analysis using computational models. In: Phelps EA, Robbins TW, Delgado M (eds) Decision making, affect, and learning: Attention and performance XXIII (Vol. XXIII). Oxford University Press. 10.1093/acprof:oso/9780199600434.003.0001.

[CR24] Delinte A, Gomez CM, Decostre MF, Crommelinck M, Roucoux A (2002) Amplitude transition function of human express saccades. Neurosci Res 42(1):21–34. 10.1016/s0168-0102(01)00300-5 11814606 10.1016/s0168-0102(01)00300-5

[CR25] Deouell LY (2007) The frontal generator of the mismatch negativity revisited. Journal of Psychophysiology 21(3–4):188–203. 10.1027/0269-8803.21.34.188

[CR26] Diener C, Struve M, Balz N, Kuehner C, Flor H (2009) Exposure to uncontrollable stress and the postimperative negative variation (PINV): prior control matters. Biol Psychol 80(2):189–195. 10.1016/j.biopsycho.2008.09.002 18838101 10.1016/j.biopsycho.2008.09.002

[CR27] Donchin E (1981) Surprise! … Surprise? Psychophysiology 18(5):493–513. 10.1111/j.1469-8986.1981.tb01815.x 7280146 10.1111/j.1469-8986.1981.tb01815.x

[CR28] Donchin E, Coles MGH (1988) Is the P300 component a manifestation of context updating? Behav Brain Sci 11(3):357–374. 10.1017/S0140525X00058027

[CR29] Donchin E, Ritter W, McCallum C (1978a) Cognitive psychophysiology: the endogenous components of the ERP. In: Callaway E, Tueting P, Koslow SH (eds) Brain Event-related Potentials in Man. Academic, New York, pp 349–411

[CR30] Donchin E, Ritter W, McCallum WC (1978b) Cognitive psychophysiology: The endogenous components of the ERP. In E. Callaway, P. Tueting, & S. H. Koslow (Eds.), Event-related brain potentials in man (pp. 349–411). Academic Press. 10.1016/B978-0-12-155150-6.50019-5

[CR31] Donchin E, Karis D, Bashore TR, Coles MGH, Gratton G (1986) Cognitive psychophysiology and human information processing. In: Coles MGH, Donchin E, Porges SW (eds) Psychophysiology: Systems, processes, and applications. Guilford Press, pp 244–267

[CR32] Dongier M (1969) Separation of the various independent phenomena among the slow potential changes (contingent negative variations). Electroencephalogr Clin Neurophysiol 27(1):108–109 4182882

[CR33] Duncan-Johnson CC, Donchin E (1977) On quantifying surprise: the variation of event-related potentials with subjective probability. Psychophysiology 14(5):456–467. 10.1111/j.1469-8986.1977.tb01312.x 905483 10.1111/j.1469-8986.1977.tb01312.x

[CR34] Duncan-Johnson CC, Donchin E (1982) The P300 component of the event-related brain potential as an index of information processing. Biol Psychol 0511(82):1–52. 10.1016/0301-0511(82)90016-3 10.1016/0301-0511(82)90016-3 6809064

[CR35] Eimer M (1993) Spatial cueing, sensory gating and selective response preparation: an ERP study on visuo-spatial orienting. Electroencephalogr Clin Neurophysiol 88(5):408–420. 10.1016/0168-5597(93)90017-j 7691565 10.1016/0168-5597(93)90017-j

[CR36] Elbert T, Rockstroh B, Lutzenberger W, Birbaumer N (1970) Slow brain potentials after withdrawal of control. Archiv für Psychiatrie und Nervenkrankheiten 232(3):201–214. 10.1007/BF02141781 10.1007/BF02141781 7159206

[CR37] Escera C, Corral MJ (2007) Role of mismatch negativity and novelty-P3 in involuntary auditory attention. Journal of Psychophysiology 21(3–4):251–264. 10.1027/0269-8803.21.34.251

[CR38] Evans JL, Saffran JR, Robe-Torres K (2009) Statistical learning in children with specific language impairment. Journal of Speech, Language, and Hearing Research 52(2):321–335. 10.1044/1092-4388(2009/07-0189) 19339700 10.1044/1092-4388(2009/07-0189) PMC3864761

[CR39] Feuerriegel D, Yook J, Quek GL, Hogendoorn H, Bode S (2021) Visual mismatch responses index surprise signalling but not expectation suppression. Cortex; a journal devoted to the study of the nervous system and behavior. 134:16–29. 10.1016/j.cortex.2020.10.006 10.1016/j.cortex.2020.10.006 33249297

[CR40] Flores AB, Digiacomo MR, Meneres S, Trigo E, Gómez CM (2009) Development of preparatory activity indexed by the contingent negative variation in children. Brain Cogn 71(2):129–140. 10.1016/j.bandc.2009.04.011 19500893 10.1016/j.bandc.2009.04.011

[CR41] Fonteneau E, van der Lely HK (2008) Electrical brain responses in language-impaired children reveal grammar-specific deficits. PLoS ONE 3(3):e1832. 10.1371/journal.pone.0001832 18347740 10.1371/journal.pone.0001832 PMC2268250

[CR42] Friedman D, Cycowicz YM, Gaeta H (2001) The novelty P3: an event-related brain potential (ERP) sign of the brain’s evaluation of novelty. Neurosci Biobehav Rev 25(4):355–373. 10.1016/s0149-7634(01)00019-7 11445140 10.1016/s0149-7634(01)00019-7

[CR43] Friston K (2005) A theory of cortical responses. Philos Trans R Soc Lond B Biol Sci 360(1456):815–836. 10.1098/rstb.2005.1622 15937014 10.1098/rstb.2005.1622 PMC1569488

[CR44] Friston K (2009) The free-energy principle: a rough guide to the brain? Trends Cogn Sci 13(7):293–301. 10.1016/j.tics.2009.04.005 19559644 10.1016/j.tics.2009.04.005

[CR45] Friston K (2010) The free-energy principle: a unified brain theory? Nat Rev Neurosci 11(2):127–138. 10.1038/nrn2787 20068583 10.1038/nrn2787

[CR46] Friston K, Kiebel S (2009) Predictive coding under the free-energy principle. Philosophical Transactions of the Royal Society B: Biological Sciences 364(1521):1211–1221. 10.1098/rstb.2008.0300 10.1098/rstb.2008.0300 PMC2666703 19528002

[CR47] Friston K, Rigoli F, Ognibene D, Mathys C, Fitzgerald T, Pezzulo G (2015) Active inference and epistemic value. Cogn Neurosci 6(4):187–214. 10.1080/17588928.2015.1020053 25689102 10.1080/17588928.2015.1020053

[CR48] Garrido MI, Kilner JM, Stephan KE, Friston KJ (2009) The mismatch negativity: a review of underlying mechanisms. Clin Neurophysiol 120(3):453–463. 10.1016/j.clinph.2008.11.029 19181570 10.1016/j.clinph.2008.11.029 PMC2671031

[CR50] Gómez RL (2002) Variability and detection of invariant structure. Psychol Sci 13(5):431–436. 10.1111/1467-9280.00476 12219809 10.1111/1467-9280.00476

[CR49] Gomez RL, Gerken L (1999) Artificial grammar learning by 1-year-olds leads to specific and abstract knowledge. Cognition 70(2):109–135. 10.1016/s0010-0277(99)00003-7 10349760 10.1016/s0010-0277(99)00003-7

[CR51] Gómez CM, Flores A (2011) A neurophysiological evaluation of a cognitive cycle in humans. Neurosci Biobehav Rev 35(3):452–461. 10.1016/j.neubiorev.2010.05.005 20685362 10.1016/j.neubiorev.2010.05.005

[CR52] Gómez CM, Delinte A, Vaquero E, Cardoso MJ, Vázquez M, Crommelinck M, Roucoux A (2001) Current source density analysis of CNV during temporal gap paradigm. Brain Topogr 13(3):149–159. 10.1023/a:1007816201345 11302395 10.1023/a:1007816201345

[CR53] Gómez CM, Marco J, Grau C (2003) Preparatory visuo-motor cortical network of the contingent negative variation estimated by current density. NeuroImage 20(1):216–224. 10.1016/s1053-8119(03)00295-7 14527582 10.1016/s1053-8119(03)00295-7

[CR54] Gómez CM, Fernández A, Maestú F, Amo C, González-Rosa JJ, Vaquero E, Ortiz T (2004) Task-specific sensory and motor preparatory activation revealed by contingent magnetic variation. Brain Res Cogn Brain Res 21(1):59–68. 10.1016/j.cogbrainres.2004.05.005 15325413 10.1016/j.cogbrainres.2004.05.005

[CR55] Gómez CM, Flores A, Digiacomo MR, Vázquez-Marrufo M (2009) Sequential P3 effects in a Posner’s spatial cueing paradigm: trial-by-trial learning of the predictive value of the cue. Acta Neurobiol Exp 69(2):155–167. 10.55782/ane-2009-1741 10.55782/ane-2009-1741 19593330

[CR56] Gómez CM, Arjona A, Donnarumma F, Maisto D, Rodríguez-Martínez EI, Pezzulo G (2019) Tracking the Time Course of Bayesian Inference With Event-Related Potentials:A Study Using the Central Cue Posner Paradigm. Front Psychol 10:1424. 10.3389/fpsyg.2019.01424 31275215 10.3389/fpsyg.2019.01424 PMC6593096

[CR57] Haebig E, Weber C, Leonard LB, Deevy P, Tomblin JB (2017) Neural patterns elicited by sentence processing uniquely characterize typical development, SLI recovery, and SLI persistence. J neurodevelopmental disorders 9:22. 10.1186/s11689-017-9201-1 10.1186/s11689-017-9201-1 PMC5470275 28630655

[CR58] Haebig E, Leonard L, Usler E, Deevy P, Weber C (2018) An Initial Investigation of the Neural Correlates of Word Processing in Preschoolers With Specific Language Impairment. Journal of speech, language, and hearing research. JSLHR 61(3):729–739. 10.1044/2017_JSLHR-L-17-0249 29484362 10.1044/2017_JSLHR-L-17-0249 PMC6195066

[CR59] Haenschel C, Vernon DJ, Dwivedi P, Gruzelier JH, Baldeweg T (2005) Event-related brain potential correlates of human auditory sensory memory-trace formation. J Neurosci 25(45):10494–10501. 10.1523/JNEUROSCI.1227-05.2005 16280587 10.1523/JNEUROSCI.1227-05.2005 PMC6725828

[CR60] Heslenfeld D (2003) Visual mismatch negativity. In: Polich J (ed) Detection of change: event-related potential and fMRI findings. Kluwer, Boston, MA, pp 41–59

[CR61] Higashi H, Minami T, Nakauchi S (2017) Variation in event-related potentials by state transitions. Front Hum Neurosci 11:75. 10.3389/fnhum.2017.00075 28289380 10.3389/fnhum.2017.00075 PMC5326784

[CR62] Hohwy J (2013) The Predictive Mind. Oxford. 10.1093/ACPROF:OSO/9780199682737.001.0001

[CR63] Hohwy J (2020) New directions in predictive processing. Mind Lang 35(2):209–223

[CR64] Howard M, Volkov IO, Abbas PJ, Damasio H, Ollendieck MC, Granner MA (1996) A chronic microelectrode investigation of the tonotopic organization of human auditory cortex. Brain Res 724(2):260–264. 10.1016/0006-8993(96)00315-0 8828578 10.1016/0006-8993(96)00315-0

[CR65] Hsu HJ, Bishop DV (2011) Grammatical difficulties in children with specific language impairment: is learning deficient? Hum Dev 53(5):264–277. 10.1159/000321289 22003258 10.1159/000321289 PMC3191529

[CR66] Hsu HJ, Tomblin JB, Christiansen MH (2014) Impaired statistical learning of non-adjacent dependencies in adolescents with specific language impairment. Front Psychol 5:175. 10.3389/fpsyg.2014.00175 24639661 10.3389/fpsyg.2014.00175 PMC3944677

[CR67] Huber L, Tse DHY, Wiggins CJ, Uludağ K, Kashyap S, Jangraw DC, Bandettini PA, Poser BA, Ivanov D (2018) Ultra-high resolution blood volume fMRI and BOLD fMRI in humans at 9.4 T: Capabilities and challenges. NeuroImage. 178:769–779. 10.1016/j.neuroimage.2018.06.025 10.1016/j.neuroimage.2018.06.025 PMC6100753 29890330

[CR68] Jääskeläinen IP, Ahveninen J, Bonmassar G, Dale AM, Ilmoniemi RJ, Levänen S, Lin FH, May P, Melcher J, Stufflebeam S, Tiitinen H, Belliveau JW (2004) Human posterior auditory cortex gates novel sounds to consciousness. Proc Natl Acad Sci USA 101(17):6809–6814. 10.1073/PNAS.0303760101 15096618 10.1073/pnas.0303760101 PMC404127

[CR69] Kathmann N, Jonitz L, Engel RR (1990) Cognitive determinants of the postimperative negative variation. Psychophysiology 27(3):256–263. 10.1111/j.1469-8986.1990.tb00380.x 2236429 10.1111/j.1469-8986.1990.tb00380.x

[CR70] Kenemans JL, Kok A, Smulders FT (1993) Event-related potentials to conjunctions of spatial frequency and orientation as a function of stimulus parameters and response requirements. Electroencephalogr Clin Neurophysiol 88(1):51–63. 10.1016/0168-5597(93)90028-n 7681391 10.1016/0168-5597(93)90028-n

[CR71] Klein C, Rockstroh B, Cohen R, Berg P, Dressel M (1996) The impact of performance uncertainty on the postimperative negative variation. Psychophysiology 33(4):426–433. 10.1111/j.1469-8986.1996.tb01068.x 8753943 10.1111/j.1469-8986.1996.tb01068.x

[CR72] Knill DC, Pouget A (2004) The Bayesian brain: The role of uncertainty in neural coding and computation. Trends Neurosci 27(12):712–719. 10.1016/j.tins.2004.10.007 15541511 10.1016/j.tins.2004.10.007

[CR73] Kok P, Bains LJ, van Mourik T, Norris DG, de Lange FP (2016) Selective Activation of the Deep Layers of the Human Primary Visual Cortex by Top-Down Feedback. Curr biology: CB 26(3):371–376. 10.1016/j.cub.2015.12.038 10.1016/j.cub.2015.12.038 26832438

[CR74] Kolossa A, Fingscheidt T, Wessel K, Kopp B (2013) A model-based approach to trial-by-trial p300 amplitude fluctuations. Front Hum Neurosci 6:359. 10.3389/fnhum.2012.00359 23404628 10.3389/fnhum.2012.00359 PMC3567611

[CR75] Kolossa A, Kopp B, Fingscheidt T (2015) A computational analysis of the neural bases of Bayesian inference. NeuroImage 106:222–237. 10.1016/j.neuroimage.2014.11.007 25462794 10.1016/j.neuroimage.2014.11.007

[CR76] Kopp B, Seer C, Lange F, Kluytmans A, Kolossa A, Fingscheidt T, Hoijtink H (2016) P300 amplitude variations, prior probabilities, and likelihoods: A Bayesian ERP study. Cogn Affect Behav Neurosci 16(5):911–928. 10.3758/s13415-016-0442-3 27406085 10.3758/s13415-016-0442-3

[CR77] Korzyukov OA, Winkler I, Gumenyuk VI, Alho K (2003) Processing abstract auditory features in the human auditory cortex. NeuroImage 20(4):2245–2258. 10.1016/j.neuroimage.2003.08.014 14683726 10.1016/j.neuroimage.2003.08.014

[CR78] Kujala T, Leminen M (2017) Low-level neural auditory discrimination dysfunctions in specific language impairment-A review on mismatch negativity findings. Dev Cogn Neurosci 28:65–75. 10.1016/j.dcn.2017.10.005 29182947 10.1016/j.dcn.2017.10.005 PMC6987907

[CR79] Kutas M, Hillyard SA (1984) Brain potentials during reading reflect word expectancy and semantic association. Nature 307(5947):161–163. 10.1038/307161a0 6690995 10.1038/307161a0

[CR80] Lang AH, Eerola O, Korpilahti P, Holopainen I, Salo S, Aaltonen O (1995) Practical issues in the clinical application of mismatch negativity. Ear Hear 16(1):118–130. 10.1097/00003446-199502000-00009 7774765 10.1097/00003446-199502000-00009

[CR81] Lauter JL, Herscovitch P, Formby C, Raichle ME (1985) Tonotopic organization in human auditory cortex revealed by positron emission tomography. Hear Res 20(3):199–205. 10.1016/0378-5955(85)90024-3 3878839 10.1016/0378-5955(85)90024-3

[CR82] León-Cabrera P, Rodríguez-Fornells A, Morís J (2017) Electrophysiological correlates of semantic anticipation during speech comprehension. Neuropsychologia 99:326–334. 10.1016/j.neuropsychologia.2017.02.026 28300582 10.1016/j.neuropsychologia.2017.02.026

[CR83] León-Cabrera P, Hjortdal A, Berthelsen SG, Rodríguez-Fornells A, Roll M (2024) Neurophysiological signatures of prediction in language: A critical review of anticipatory negativities. Neurosci Biobehav Rev 160:105624. 10.1016/j.neubiorev.2024.105624 38492763 10.1016/j.neubiorev.2024.105624

[CR84] Lieder F, Daunizeau J, Garrido MI, Friston KJ, Stephan KE (2013) Modelling trial-by-trial changes in the mismatch negativity. PLoS Comput Biol 9(2):e1002911. 10.1371/journal.pcbi.1002911 23436989 10.1371/journal.pcbi.1002911 PMC3578779

[CR85] Løvstad M, Funderud I, Endestad T, Due-Tønnessen P, Meling TR, Lindgren M, Knight RT, Solbakk AK (2012) Executive functions after orbital or lateral prefrontal lesions: neuropsychological profiles and self-reported executive functions in everyday living. Brain Injury 26(13–14):1586–1598. 10.3109/02699052.2012.698787 22731818 10.3109/02699052.2012.698787 PMC4090100

[CR86] Luck SJ (2014) An introduction to the event-related potential technique, 2nd edn. MIT Press

[CR87] Lum JA, Conti-Ramsden G, Page D, Ullman MT (2012) Working, declarative and procedural memory in specific language impairment. Cortex 48(9):1138–1154. 10.1016/j.cortex.2011.06.001 21774923 10.1016/j.cortex.2011.06.001 PMC3664921

[CR88] Lum JA, Conti-Ramsden G, Morgan AT, Ullman MT (2014) Procedural learning deficits in specific language impairment (SLI): a meta-analysis of serial reaction time task performance. Cortex 51(100):1–10. 10.1016/j.cortex.2013.10.011 24315731 10.1016/j.cortex.2013.10.011 PMC3989038

[CR89] Mainela-Arnold E, Evans JL (2014) Do statistical segmentation abilities predict lexical-phonological and lexical-semantic abilities in children with and without SLI? J Child Lang 41(2):327–351. 10.1017/S0305000912000736 23425593 10.1017/S0305000912000736 PMC4083839

[CR90] Makeig S, Westerfield M, Jung TP, Covington J, Townsend J, Sejnowski TJ, Courchesne E (1999) Functionally independent components of the late positive event-related potential during visual spatial attention. J Neurosci 19(7):2665–2680. 10.1523/JNEUROSCI.19-07-02665.1999 10087080 10.1523/JNEUROSCI.19-07-02665.1999 PMC6786079

[CR91] Mangun GR, Hillyard SA (1991) Modulations of sensory-evoked brain potentials indicate changes in perceptual processing during visual-spatial priming. Journal of experimental psychology. Hum Percept Perform 17(4):1057–1074. 10.1037/0096-1523.17.4.1057 10.1037//0096-1523.17.4.1057 1837297

[CR92] Marco-Pallarés J, Münte TF, Rodríguez-Fornells A (2015) The role of high-frequency oscillatory activity in reward processing and learning. Neurosci Biobehav Rev 49:1–7. 10.1016/j.neubiorev.2014.11.014 25464028 10.1016/j.neubiorev.2014.11.014

[CR93] Martikainen MH, Kaneko K, Hari R (2005) Suppressed responses to self-triggered sounds in the human auditory cortex. Cereb cortex (New York N Y: 1991) 15(3):299–302. 10.1093/cercor/bhh131 10.1093/cercor/bhh131 15238430

[CR94] Mathys C, Daunizeau J, Friston KJ, Stephan KE (2011) A bayesian foundation for individual learning under uncertainty. Front Hum Neurosci 5:39. 10.3389/fnhum.2011.00039 21629826 10.3389/fnhum.2011.00039 PMC3096853

[CR95] May PJ, Tiitinen H (2004) The MMN is a derivative of the auditory N100 response. *Neurology & clinical neurophysiology*: NCN, 2004, 20 16012601

[CR96] May PJ, Tiitinen H (2010) Mismatch negativity (MMN), the deviance-elicited auditory deflection, explained. Psychophysiology 47(1):66–122. 10.1111/j.1469-8986.2009.00856.x 19686538 10.1111/j.1469-8986.2009.00856.x

[CR97] May P, Tiitinen H, Ilmoniemi RJ, Nyman G, Taylor JG, Näätänen R (1999) Frequency change detection in human auditory cortex. J Comput Neurosci 6(2):99–120. 10.1023/a:1008896417606 10333158 10.1023/a:1008896417606

[CR98] Mendonça D, Curado M, S. Gouveia S (2020) The Philosophy and Science of Predictive Processing. 1–234. 10.5040/9781350099784

[CR99] Mento G (2013) The passive CNV: carving out the contribution of task-related processes to expectancy. Front Hum Neurosci 7:827. 10.3389/fnhum.2013.00827 24376409 10.3389/fnhum.2013.00827 PMC3859886

[CR100] Michaelov JA, Bardolph MD, Van Petten CK, Bergen BK, Coulson S (2024) Strong Prediction: Language Model Surprisal Explains Multiple N400 Effects. Neurobiology of language (Cambridge, Mass. 5(1):107–135. 10.1162/nol_a_00105 10.1162/nol_a_00105 PMC11025652 38645623

[CR101] Misyak JB, Christiansen MH (2012) Statistical learning and language: An individual differences study. Lang Learn 62(1):302–331. 10.1111/j.1467-9922.2010.00626.x

[CR102] Muñoz-Caracuel M, Muñoz V, Ruiz-Martínez FJ, Vázquez Morejón AJ, Gómez CM (2024) Systemic neurophysiological signals of auditory predictive coding. Psychophysiology 61(6):e14544. 10.1111/psyp.14544 38351668 10.1111/psyp.14544

[CR103] Näätänen R (1995) The mismatch negativity: A powerful tool for cognitive neuroscience. Ear Hear 16:6–18 7774770

[CR104] Näätänen R, Winkler I (1999) The concept of auditory stimulus representation in neuroscience. Psychol Bulleting 125:826–859 10.1037/0033-2909.125.6.826 10589304

[CR105] Näätänen R, Paavilainen P, Rinne T, Alho K (2007) The mismatch negativity (MMN) in basic research of central auditory processing: a review. Clin Neurophysiol 118(12):2544–2590. 10.1016/j.clinph.2007.04.026 17931964 10.1016/j.clinph.2007.04.026

[CR106] Näätänen R, Kujala T, Escera C, Baldeweg T, Kreegipuu K, Carlson S, Ponton C (2012) The mismatch negativity (MMN)--a unique window to disturbed central auditory processing in ageing and different clinical conditions. Clin Neurophysiol 123(3):424–458. 10.1016/j.clinph.2011.09.020 22169062 10.1016/j.clinph.2011.09.020

[CR107] Novitski N, Huotilainen M, Tervaniemi M, Näätänen R, Fellman V (2007) Neonatal frequency discrimination in 250-4000-Hz range: electrophysiological evidence. Clin Neurophysiol 118(2):412–419. 10.1016/j.clinph.2006.10.008 17134940 10.1016/j.clinph.2006.10.008

[CR108] Obeid R, Brooks PJ, Powers KL, Gillespie-Lynch K, Lum JA (2016) Statistical learning in specific language impairment and autism spectrum disorder: a meta-analysis. Front Psychol 7:1245. 10.3389/fpsyg.2016.01245 27602006 10.3389/fpsyg.2016.01245 PMC4993848

[CR109] Ono K, Yamasaki D, Altmann CF, Mima T (2017) The effect of illusionary perception on mismatch negativity (MMN): An electroencephalography study. Hear Res 356:87–92. 10.1016/j.heares.2017.10.006 29074265 10.1016/j.heares.2017.10.006

[CR110] Paavilainen P (2013) The mismatch-negativity (MMN) component of the auditory event-related potential to violations of abstract regularities: a review. Int J Psychophysiol 88(2):109–123. 10.1016/j.ijpsycho.2013.03.015 23542165 10.1016/j.ijpsycho.2013.03.015

[CR111] Paavilainen P, Saarinen J, Tervaniemi M, Näätänen R (1995) Mismatch negativity to changes in abstract sound features during dichotic listening. J Psychophysiol 9:243–249

[CR112] Paavilainen P, Jaramillo M, Näätänen R (1998) Binaural information can converge in abstract memory traces. Psychophysiology 35(5):483–487. 10.1017/s0048577298970895 9715092 10.1017/s0048577298970895

[CR113] Paavilainen P, Jaramillo M, Näätänen R, Winkler I (1999) Neuronal populations in the human brain extracting invariant relationships from acoustic variance. Neurosci Lett 265(3):179–182. 10.1016/s0304-3940(99)00237-2 10327160 10.1016/s0304-3940(99)00237-2

[CR114] Paavilainen P, Kaukinen C, Koskinen O, Kylmälä J, Rehn L (2018) Mismatch negativity (MMN) elicited by abstract regularity violations in two concurrent auditory streams. Heliyon 4(4):e00608. 10.1016/j.heliyon.2018.e00608 29862369 10.1016/j.heliyon.2018.e00608 PMC5968198

[CR115] Pantev C, Hoke M, Lehnertz K, Lütkenhöner B, Anogianakis G, Wittkowski W (1988) Tonotopic organization of the human auditory cortex revealed by transient auditory evoked magnetic fields. Electroencephalogr Clin Neurophysiol 69(2):160–170. 10.1016/0013-4694(88)90211-8 2446835 10.1016/0013-4694(88)90211-8

[CR116] Parisi G, Mazzi C, Colombari E, Mele S, Savazzi S (2026) Contextual Updating in Attentional Orienting Relies on the Right Temporoparietal Junction: Evidence From rTMS. Eur J Neurosci 63(4):e70429. 10.1111/ejn.70429 41720629 10.1111/ejn.70429 PMC12923352

[CR117] Picton TW (1988) The endogenous evoked potentials. In E. Başar (Ed.), Dynamics of sensory and cognitive processing by the brain. Springer. 10.1007/978-3-642-71531-0_17

[CR118] Pinotsis DA, Loonis R, Bastos AM, Miller EK, Friston KJ (2019) Bayesian Modelling of Induced Responses and Neuronal Rhythms. Brain Topogr 32(4):569–582. 10.1007/s10548-016-0526-y 27718099 10.1007/s10548-016-0526-y PMC6592965

[CR119] Plante E, Ogilvie T, Vance R, Aguilar JM, Dailey NS, Meyers C, Lieser AM, Burton R (2014) Variability in the language input to children enhances learning in a treatment context. Am J speech-language Pathol 23(4):530–545. 10.1044/2014_AJSLP-13-0038 10.1044/2014_AJSLP-13-0038 24700145

[CR120] Polich J (2007) Updating P300: an integrative theory of P3a and P3b. Clin Neurophysiol 118(10):2128–2148. 10.1016/j.clinph.2007.04.019 17573239 10.1016/j.clinph.2007.04.019 PMC2715154

[CR121] Polich J, Eischen SE, Collins GE (1994) P300 from a single auditory stimulus. Electroencephalogr Clin Neurophysiol 92(3):253–261. 10.1016/0168-5597(94)90068-x 7514994 10.1016/0168-5597(94)90068-x

[CR122] Potts GF, Tucker DM (2001) Frontal evaluation and posterior representation in target detection. Brain Res Cogn Brain Res 11(1):147–156. 10.1016/s0926-6410(00)00075-6 11240117 10.1016/s0926-6410(00)00075-6

[CR123] Potts GF, Dien J, Hartry-Speiser AL, McDougal LM, Tucker DM (1998) Dense sensor array topography of the event-related potential to task-relevant auditory stimuli. Electroencephalogr Clin Neurophysiol 106(5):444–456. 10.1016/s0013-4694(97)00160-0 9680158 10.1016/s0013-4694(97)00160-0

[CR124] Potts GF, Patel SH, Azzam PN (2004) Impact of instructed relevance on the visual ERP. Int J Psychophysiol 52(2):197–209. 10.1016/j.ijpsycho.2003.10.005 15050377 10.1016/j.ijpsycho.2003.10.005

[CR125] Pouget A, Beck JM, Ma WJ, Latham PE (2013) Probabilistic brains: knowns and unknowns. Nat Neurosci 16(9):1170–1178. 10.1038/nn.3495 23955561 10.1038/nn.3495 PMC4487650

[CR126] Pritchard W (1981) Psychophysiology of P300. Psychol Bull 89(3):506–540. 10.1037/0033-2909.89.3.506 7255627

[CR127] Purdy JD, Leonard LB, Weber-Fox C, Kaganovich N (2014) Decreased sensitivity to long-distance dependencies in children with a history of specific language impairment: electrophysiological evidence. Journal of speech, language, and hearing research. JSLHR 57(3):1040–1059. 10.1044/2014_JSLHR-L-13-0176 24686983 10.1044/2014_JSLHR-L-13-0176 PMC4433008

[CR128] Rabinovich E, Telesheva K (2025) The neurophysiological features of anticipation in schizophrenia: a cross-sectional study of event-related potentials. Consortium psychiatricum 6(2):21–34. 10.17816/CP15558 40927659 10.17816/CP15558 PMC12416553

[CR129] Rao RP, Ballard DH (1999) Predictive coding in the visual cortex: a functional interpretation of some extra-classical receptive-field effects. Nat Neurosci 2(1):79–87. 10.1038/4580 10195184 10.1038/4580

[CR130] Rockstroh B, Elbert T, Birbaumer N, Lutzenberger W (1982) Slow Brain Potentials and Behavior. Urban & Schwarzenberg, Baltimore, MD, USA

[CR131] Romani GL, Williamson SJ, Kaufman L (1982) Tonotopic organization of the human auditory cortex. Science 216(4552):1339–1340. 10.1126/science.7079770 7079770 10.1126/science.7079770

[CR132] Royle P, Courteau E (2014) Language processing in children with specific language impairment: A review of event-related potential studies. Language Processing: New Research. Nova Science

[CR133] Ruiz-Martínez FJ, Arjona A, Gómez CM (2021) Mismatch negativity and stimulus-preceding negativity in paradigms of increasing auditory complexity: a possible role in predictive coding. Entropy 23(3):346. 10.3390/e23030346 33804068 10.3390/e23030346 PMC7999243

[CR134] Ruiz-Martínez FJ, Morales-Ortiz M, Gómez CM (2022) Late N1 and postimperative negative variation analysis depending on the previous trial history in paradigms of increasing auditory complexity. J Neurophysiol 127(5):1240–1252. 10.1152/jn.00313.2021 35389770 10.1152/jn.00313.2021

[CR135] Ruiz-Martínez FJ, Muñoz-Caracuel M, Muñoz V, Treviño AG, Gómez CM (2025) Event-Related Spectral Perturbations differences analyzed in standard-deviant tone sequences presented in passive and active conditions. Neuroscience 571:19–30. 10.1016/j.neuroscience.2025.02.020 39993666 10.1016/j.neuroscience.2025.02.020

[CR136] Sabisch B, Hahne A, Glass E, von Suchodoletz W, Friederici AD (2006) Lexical-semantic processes in children with specific language impairment. NeuroReport 17(14):1511–1514. 10.1097/01.wnr.0000236850.61306.91 16957599 10.1097/01.wnr.0000236850.61306.91

[CR137] Saffran JR, Newport EL, Aslin RN, Tunick RA, Barrueco S (1997) Incidental language learning: Listening (and learning) out of the corner of your ear. Psychol Sci 8(2):101–105. 10.1111/j.1467-9280.1997.tb00690.x

[CR138] Seer C, Lange F, Boos M, Dengler R, Kopp B (2016) Prior probabilities modulate cortical surprise responses: A study of event-related potentials. Brain Cogn 106:78–89. 10.1016/j.bandc.2016.04.011 27266394 10.1016/j.bandc.2016.04.011

[CR139] Shafer VL, Morr ML, Datta H, Kurtzberg D, Schwartz RG (2005) Neurophysiological indexes of speech processing deficits in children with specific language impairment. J Cogn Neurosci 17(7):1168–1180. 10.1162/0898929054475217 16138434 10.1162/0898929054475217

[CR140] Shafer VL, Ponton C, Datta H, Morr ML, Schwartz RG (2007) Neurophysiological indices of attention to speech in children with specific language impairment. Clin neurophysiology: official J Int Federation Clin Neurophysiol 118(6):1230–1243. 10.1016/j.clinph.2007.02.023 10.1016/j.clinph.2007.02.023 PMC2020430 17452008

[CR141] Shannon CE (1948) A mathematical theory of communication. Bell Syst Tech J 27:379–423. 10.1002/j.1538-7305.1948.tb01338.x

[CR142] Sommer W, Leuthold H, Matt J (1998) The expectancies that govern the P300 amplitude are mostly automatic and unconscious. Behav Brain Sci 21(1):149–150. 10.1017/S0140525X98210958

[CR143] Squires KC, Wickens C, Squires NK, Donchin E (1976) The effect of stimulus sequence on the waveform of the cortical event-related potential. Science 193(4258):1142–1146. 10.1126/science.959831 959831 10.1126/science.959831

[CR144] Summerfield C, de Lange FP (2014) Expectation in perceptual decision making: neural and computational mechanisms. Nat Rev Neurosci 15(11):745–756. 10.1038/nrn3838 25315388 10.1038/nrn3838

[CR145] Sussman ES (2007) A new view on the MMN and attention debate. J Psychophysiol 21(3–4):164–175. 10.1027/0269-8803.21.34.164

[CR146] Sutton S, Braren M, Zubin J, John ER (1965) Evoked-potential correlates of stimulus uncertainty. Science 150(3700):1187–1188. 10.1126/science.150.3700.1187 5852977 10.1126/science.150.3700.1187

[CR148] Tecce JJ (1972) Contingent negative variation (CNV) and psychological processes in man. Psychol Bull 77(2):73–108. 10.1037/h0032177 4621420 10.1037/h0032177

[CR149] Tervaniemi M, Maury S, Näätänen R (1994) Neural representations of abstract stimulus features in the human brain as reflected by the mismatch negativity. NeuroReport 5(7):844–846. 10.1097/00001756-199403000-00027 8018861 10.1097/00001756-199403000-00027

[CR150] Tervaniemi M, Schröger E, Saher M, Näätänen R (2000) Effects of spectral complexity and sound duration on automatic complex-sound pitch processing in humans - a mismatch negativity study. Neurosci Lett 290(1):66–70. 10.1016/s0304-3940(00)01290-8 10925176 10.1016/s0304-3940(00)01290-8

[CR151] Ullman MT, Pierpont EI (2005) Specific language impairment is not specific to language: the procedural deficit hypothesis. Cortex; a journal devoted to the study of the nervous system and behavior. 41(3):399–433. 10.1016/s0010-9452(08)70276-4 10.1016/s0010-9452(08)70276-4 15871604

[CR152] Uran C, Peter A, Lazar A, Barnes W, Klon-Lipok J, Shapcott KA, Roese R, Fries P, Singer W, Vinck M (2022) Predictive coding of natural images by V1 firing rates and rhythmic synchronization. Neuron 110(7):1240–1257. 10.1016/j.neuron.2022.01.002 35120628 10.1016/j.neuron.2022.01.002 PMC8992798

[CR153] van Herten M, Kolk HH, Chwilla DJ (2005) An ERP study of P600 effects elicited by semantic anomalies. Brain Res Cogn Brain Res 22(2):241–255. 10.1016/j.cogbrainres.2004.09.002 15653297 10.1016/j.cogbrainres.2004.09.002

[CR154] Vossel S, Mathys C, Daunizeau J, Bauer M, Driver J, Friston KJ, Stephan KE (2014) Spatial attention, precision, and Bayesian inference: a study of saccadic response speed. Cereb Cortex 24(6):1436–1450. 10.1093/cercor/bhs418 23322402 10.1093/cercor/bhs418 PMC4014178

[CR155] Wacongne C, Changeux JP, Dehaene S (2012) A neuronal model of predictive coding accounting for the mismatch negativity. J Neurosci 32(11):3665–3678. 10.1523/JNEUROSCI.5003-11.2012 22423089 10.1523/JNEUROSCI.5003-11.2012 PMC6703454

[CR156] Walsh KS, McGovern DP, Clark A, O’Connell RG (2020) Evaluating the neurophysiological evidence for predictive processing as a model of perception. Ann N Y Acad Sci 1464(1):242–268. 10.1111/nyas.14321 32147856 10.1111/nyas.14321 PMC7187369

[CR157] Walter WG, Cooper R, Aldridge VJ, McCallum WC, Winter AL (1964) Contingent Negative Variation: an electric sign of sensorimotor association and expectancy in the human brain. Nature 203(4943):380–384. 10.1038/203380A0 14197376 10.1038/203380a0

[CR158] Wang L, Schoot L, Brothers T, Alexander E, Warnke L, Kim M, Khan S, Hämäläinen M, Kuperberg GR (2023) Predictive coding across the left fronto-temporal hierarchy during language comprehension. Cereb Cortex 33(8):4478–4497. 10.1093/cercor/bhac356 36130089 10.1093/cercor/bhac356 PMC10110445

[CR159] Wetzel N, Schröger E (2014) On the development of auditory distraction: A review. PsyCh J 3(1):72–91. 10.1002/pchj.49 26271640 10.1002/pchj.49

[CR160] Winkler I (2007) Interpreting the mismatch negativity (MMN). J Psychophysiol 21:147–163. 10.1027/0269-8803.21.34.147

[CR161] Winkler I, Czigler I (2012) Evidence from auditory and visual event-related potential (ERP) studies of deviance detection (MMN and vMMN) linking predictive coding theories and perceptual object representations. Int J Psychophysiol 83(2):132–143. 10.1016/j.ijpsycho.2011.10.001 22047947 10.1016/j.ijpsycho.2011.10.001

[CR162] Woldorff MG, Hillyard SA (1991) Modulation of early auditory processing during selective listening to rapidly presented tones. Electroencephalogr Clin Neurophysiol 79(3):170–191. 10.1016/0013-4694(91)90136-r 1714809 10.1016/0013-4694(91)90136-r

[CR163] Ylinen S, Shestakova A, Huotilainen M, Alku P, Näätänen R (2006) Mismatch negativity (MMN) elicited by changes in phoneme length: a cross-linguistic study. Brain Res 1072(1):175–185. 10.1016/j.brainres.2005.12.004 16426584 10.1016/j.brainres.2005.12.004

